# Quinolinic acid potentially links kidney injury to brain toxicity

**DOI:** 10.1172/jci.insight.180229

**Published:** 2025-02-13

**Authors:** Afaf Saliba, Subrata Debnath, Ian Tamayo, Hak Joo Lee, Nagarjunachary Ragi, Falguni Das, Richard Montellano, Jana Tumova, Meyer Maddox, Esmeralda Trevino, Pragya Singh, Caitlyn Fastenau, Soumya Maity, Guanshi Zhang, Leila Hejazi, Manjeri A. Venkatachalam, Jason C. O’Connor, Bernard Fongang, Sarah C. Hopp, Kevin F. Bieniek, James D. Lechleiter, Kumar Sharma

**Affiliations:** 1 Center for Precision Medicine and; 2 Division of Nephrology, Department of Medicine, The University of Texas Health Science Center at San Antonio, San Antonio, Texas, USA.; 3 Department of Physiology, Faculty of Medicine in Pilsen, Charles University, Pilsen, Czech Republic.; 4 Department of Pharmacology,; 5 Glenn Biggs Institute for Alzheimer’s and Neurodegenerative Diseases, and; 6 Department of Pathology and Laboratory Medicine, The University of Texas Health Science Center at San Antonio, San Antonio, Texas, USA.; 7 South Texas Veterans Health Care System, Audie L. Murphy VA Hospital, San Antonio, Texas, USA.; 8 Department of Biochemistry and Structural Biology,; 9 Department of Population Health Sciences, and; 10 Department of Cell Systems and Anatomy, The University of Texas Health Science Center at San Antonio, San Antonio, Texas, USA.

**Keywords:** Nephrology, Neuroscience, Amino acid metabolism, Chronic kidney disease, Neurological disorders

## Abstract

Kidney dysfunction often leads to neurological impairment, yet the complex kidney-brain relationship remains elusive. We employed spatial and bulk metabolomics to investigate a mouse model of rapid kidney failure induced by mouse double minute 2 (*Mdm2*) conditional deletion in the kidney tubules to interrogate kidney and brain metabolism. Pathway enrichment analysis of a focused plasma metabolomics panel pinpointed tryptophan metabolism as the most altered pathway with kidney failure. Spatial metabolomics showed toxic tryptophan metabolites in the kidneys and brains, revealing a connection between advanced kidney disease and accelerated kynurenine degradation. In particular, the excitotoxic metabolite quinolinic acid was localized in ependymal cells in the setting of kidney failure. These findings were associated with brain inflammation and cell death. Separate mouse models of ischemia-induced acute kidney injury and adenine-induced chronic kidney disease also exhibited systemic inflammation and accumulating toxic tryptophan metabolites. Patients with advanced chronic kidney disease (stage 3b-4 and stage 5) similarly demonstrated elevated plasma kynurenine metabolites, and quinolinic acid was uniquely correlated with fatigue and reduced quality of life. Overall, our study identifies the kynurenine pathway as a bridge between kidney decline, systemic inflammation, and brain toxicity, offering potential avenues for diagnosis and treatment of neurological issues in kidney disease.

## Abstract 2

Metabolomics unveils toxic tryptophan metabolite linking kidney decline to toxic brain milieu.

## Graphical abstract

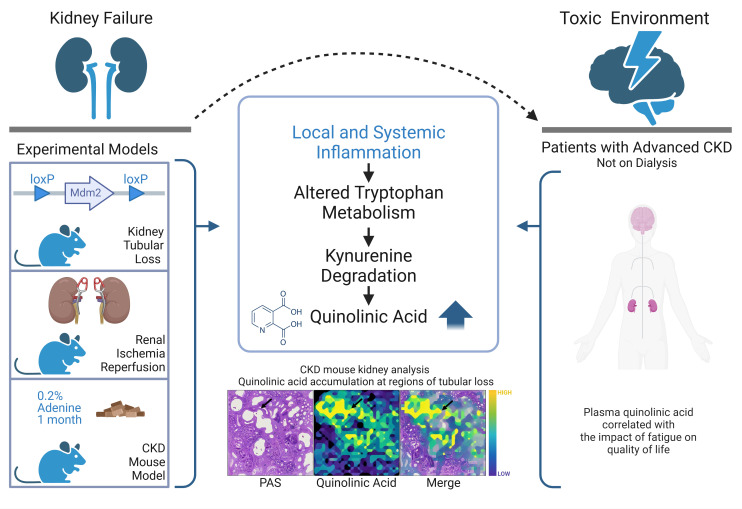

## Introduction

Acute kidney disease and chronic kidney disease (CKD), characterized by decline in kidney function, are global health burdens and can have a major impact on neurologic dysfunction (1). In the United States, acute kidney injury (AKI) has a prevalence of 7% in hospitalized patients and is associated with increased mortality risk (2, 3), and CKD has a prevalence of 14% (4) and also is associated with increased mortality (5). Patients with AKI and CKD are more prone to complications, including cardiovascular, metabolic, neurologic, and other disturbances (6). Fatigue and depression are common neuropsychiatric syndromes patients with CKD experience as kidney function progresses to stage 4 or 5 (7); however, the basis for these symptoms is unclear.

Kidney-related neurologic dysfunction has been associated with ischemic cerebrovascular lesions (8), white matter lesions (9), microbleeds (10), and circulating uremic toxins (11, 12). However, the underlying mechanisms affecting the kidney/brain axis remain poorly understood. To interrogate how reduced kidney function may lead to neurologic dysfunction, we analyzed candidate molecules via a targeted metabolomics analysis of 32 metabolites in 2 separate mouse models of rapid kidney decline and a CKD mouse model, followed by spatial metabolomics in kidney and brain. In addition, clinical translational studies were performed in patients with stage 4 and stage 5 CKD.

We found accumulation of circulating toxic metabolites produced by the catabolism of essential amino acid tryptophan (Trp) via the kynurenine pathway (KP) in the plasma, as well as in kidney and brain tissues. Clinical samples demonstrated accumulation of the same neurotoxic metabolites in plasma samples in patients with advanced CKD. In both preclinical and clinical studies, the neurotoxic metabolite quinolinic acid (QA) emerged as a key link in the kidney/brain axis.

## Results

### Trp metabolism is altered in plasma of mice with rapid kidney failure.

Based on previous studies highlighting the role of mouse double minute 2 (*Mdm2*) in human and experimental kidney disease (13, 14), we studied rapid kidney failure in the doxycycline-induced conditional knockout of *Mdm2* targeting renal tubular epithelial cells (*Mdm2*-cKO). As depicted in Supplemental Figure 1 (supplemental material available online with this article; https://doi.org/10.1172/jci.insight.180229DS1), mice with inducible kidney tubular *Mdm2* deficiency exhibited pronounced kidney dysfunction within 3–7 days. This dysfunction was marked by severe tubular cell damage and a marked decline in kidney function, evidenced by elevated blood urea nitrogen (BUN) and plasma creatinine levels. To exclude the possibility of multiple-organ failure, we conducted blood assessments of liver function markers, albumin (ALB) and alanine aminotransferase (ALT), in control versus *Mdm2*-cKO mice, showing no significant changes. Furthermore, histological analysis showed no significant differences in the morphology of the heart, lung, or liver tissues between control and *Mdm2*-cKO groups (Supplemental Figure 2).

**Figure 1:**
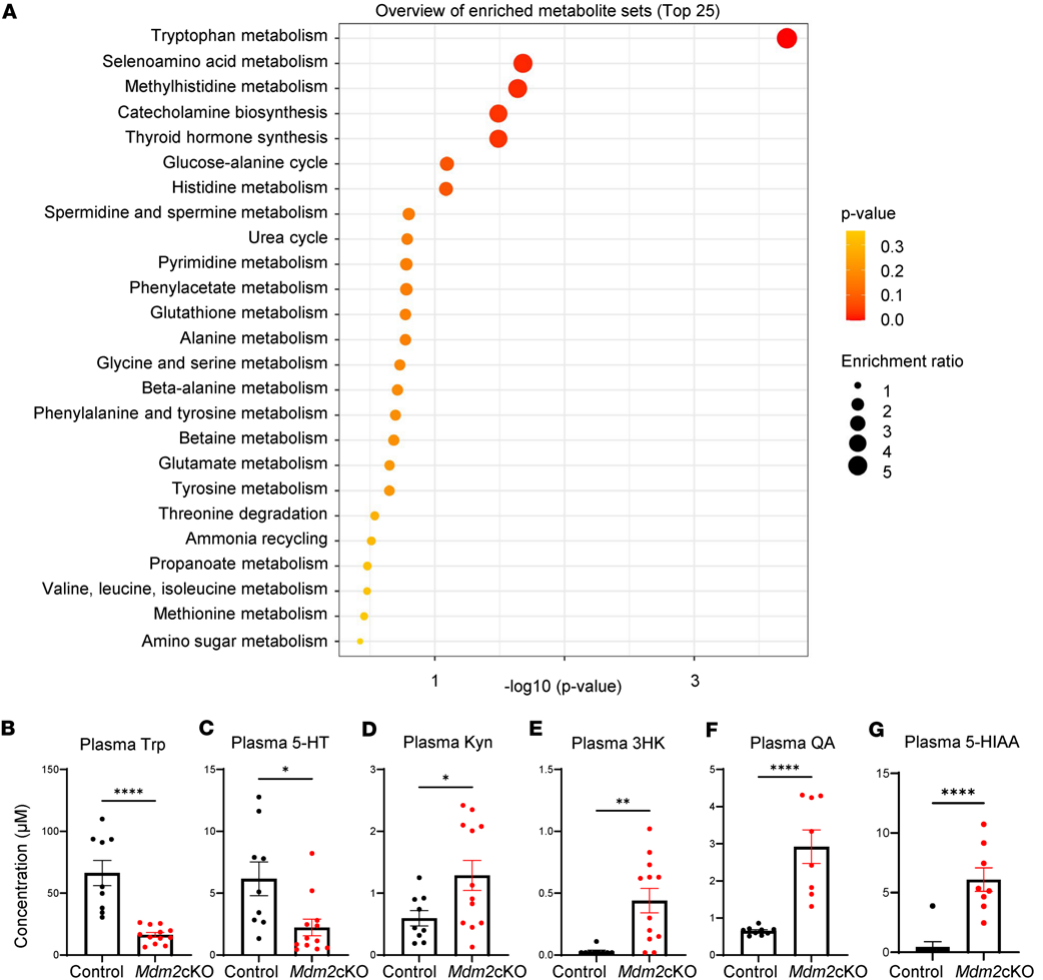
Plasma metabolomics highlight tryptophan metabolism in mice with rapid kidney failure. Targeted metabolomics incorporating 32 metabolites of the amino acid panel was performed in plasma from *Mdm2*-cKO (*n* = 12) vs. control mice (*n* = 9), at day 6 of doxycycline administration. (**A**) Overview of unbiased pathway enrichment analysis on MetaboAnalyst 5.0 (library: Small Molecule Pathway Database [SMPDB]). Raw concentrations of (**B**) tryptophan (Trp), (**C**) serotonin (5-HT), (**D**) kynurenine (KYN), (**E**) 3-hydroxykynurenine (3HK), (**F**) quinolinic acid (QA), and (**G**) 5-hydroxyindoleacetic acid (5-HIAA). QA and 5-HIAA were measured with another mass spectrometry method (*Mdm2*-cKO *n* = 8; control *n* = 9). Graphs display means ± SEM. Two-tailed *t* tests: *P < 0.05, **P < 0.01, and ****P < 0.0001.

We performed targeted bulk metabolomics in plasma samples from *Mdm2*-cKO (*n* = 12) versus control (*n* = 9) mice, at day 6 of doxycycline administration, with a panel of 32 amino acid–related metabolites relevant to human kidney disease, based on a prior untargeted metabolomics analysis from over 1,000 patients (15). Unbiased pathway enrichment analyses of the targeted panel revealed Trp metabolism as the top enriched pathway with the lowest false discovery rate (FDR) value (*P* < 0.01) (Figure 1A and Supplemental Table 1). Trp pathway metabolites in the plasma of *Mdm2*-cKO mice showed a significant decrease in Trp and serotonin (5-HT) and an increase in kynurenine (KYN), 3-hydroxykynurenine (3HK), QA, and 5-hydroxyindoleacetic acid (5-HIAA) (Figure 1, B–G). These changes indicate enhanced 5-HT degradation, explaining the low 5-HT levels as well as accelerated KYN degradation via the KP, explaining the accumulation of 3HK and QA. KP is typically activated by enzymes such as indoleamine 2,3-dioxygenase (IDO) and kynurenine 3-monooxygenase (KMO), which are often upregulated in response to inflammatory cytokines. Interestingly, the plasma KYN-to-Trp ratio, an indicator of IDO activity, and the plasma 3HK-to-KYN ratio, an indicator of KMO activity, were significantly correlated with plasma creatinine and with BUN levels from day 3 to day 6 of doxycycline administration (KYN/Trp vs. plasma creatinine: *r* = 0.86; 3HK/KYN vs. plasma creatinine: *r* = 0.85; and KYN/Trp vs. BUN: *r* = 0.92; 3HK/KYN vs. BUN: *r* = 0.82; *P* < 0.001) (Supplemental Figure 3), indicating a strong link between altered Trp metabolism and kidney function.

### Kidney failure is associated with increased inflammation and altered Trp metabolism in mice.

**Figure 2:**
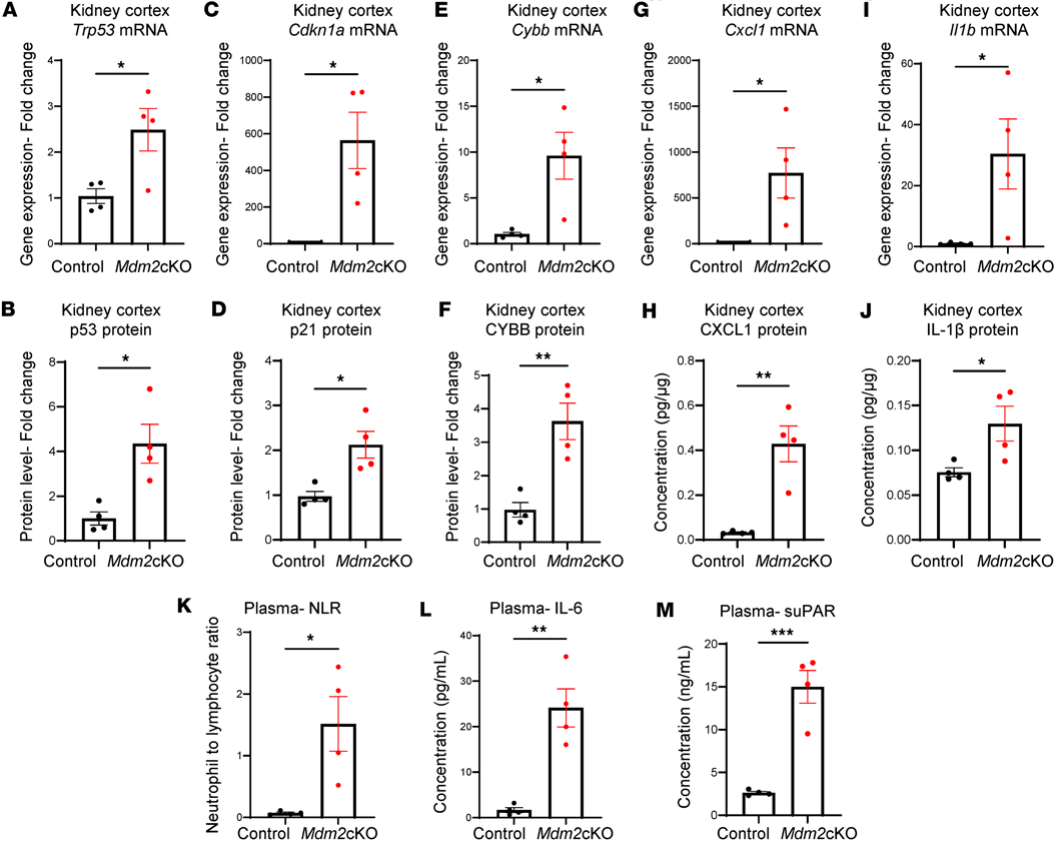
Tubular kidney cell death is associated with increased inflammation locally at the kidney cortex and at the systemic level. Kidney cortex RNA and protein lysates were prepared from *Mdm2*-cKO vs. control mice (*n* = 4 per group). (**A**) Transformation related protein 53 (*Trp53*) mRNA and (**B**) p53 protein levels. (**C**) Cyclin-dependent kinase inhibitor 1A (*Cdkn1a*) mRNA and (**D**) p21 protein levels. (**E**) Cytochrome b-245, beta polypeptide (*Cybb*), mRNA and (**F**) CYBB protein levels. (**G**) CXC chemokine ligand 1 (*Cxcl1*) mRNA level. (**H**) CXCL1 protein concentration. (**I**) Interleukin-1β (*Il1b*) mRNA level. (**J**) IL-1β protein concentration. (**K**) Neutrophil-to-lymphocyte ratio (VetScan Analyzer). (**L**) Plasma interleukin-6 (IL-6) protein concentration. (**M**) Plasma soluble urokinase plasminogen activator receptor (suPAR) concentration. Quantitative PCR (qPCR) results (normalized to *Gapdh* expression) and protein levels measured via Western blot (normalized to actin) are presented as fold-change relative to control. The proteins analyzed via ELISA are reported as concentrations. Graphs display means ± SEM. Two-tailed *t* tests: *P < 0.05, **P < 0.01, and ***P < 0.001.

*Mdm2*-cKO mice also exhibited an increase in the mRNA and protein levels of transformation related protein 53 (*Trp53*) (protein: p53), alongside the downstream target of p53, cyclin-dependent kinase inhibitor 1A (*Cdkn1a*) (protein: p21), indicating a response to cell stress. In addition, there was a significant increase in the mRNA and protein levels of cytochrome b-245 heavy chain (*Cybb*) (protein: CYBB; also known as NADPH oxidase or NOX2), indicating stimulation of oxidative stress with kidney failure (Figure 2, A–F, and Supplemental Figure 1, B and C). Despite the increase in mRNA level of hypoxia-inducible factor 1, α subunit, HIF-1α protein levels were decreased (Supplemental Figure 4, A and B), implying potential posttranscriptional regulation, possibly through ubiquitin-mediated degradation or altered protein stability in a hypoxic environment.

Moreover, several inflammatory markers were measured. Those that were significantly upregulated at the mRNA and protein levels are CXC chemokine ligand 1 (CXCL1), which mediates migration of immune cells to the inflammation site, and the cytokine interleukin-1β (IL-1β) (Figure 2, G–J). CC chemokine ligand 2 (CCL2), which recruits monocytes, showed a trend of increase at both mRNA and protein levels, while interleukin-6 (*Il6*) exhibited a trend of increase at the mRNA level without significant changes in IL-6 protein levels (Supplemental Figure 4, C–F).

As kidney inflammation can contribute to systemic inflammation through the release of inflammatory mediators (16), we found an elevated neutrophil-to-lymphocyte ratio in the *Mdm2*-cKO mice compared with the control group (*P* = 0.02) (Figure 2K) and a significant increase in plasma IL-6 protein levels (Figure 2L). Given the presence of soluble urokinase plasminogen activator receptor (suPAR) in the serum and cerebrospinal fluid (CSF) of patients with neurological diseases (17–19), and the demonstrated role of suPAR in AKI and inflammation (20), we measured plasma suPAR levels and found them significantly elevated in the *Mdm2*-cKO group (Figure 2M). Our data indicate systemic inflammation in mice because of severe kidney tubular cell death and kidney dysfunction.

**Figure 3:**
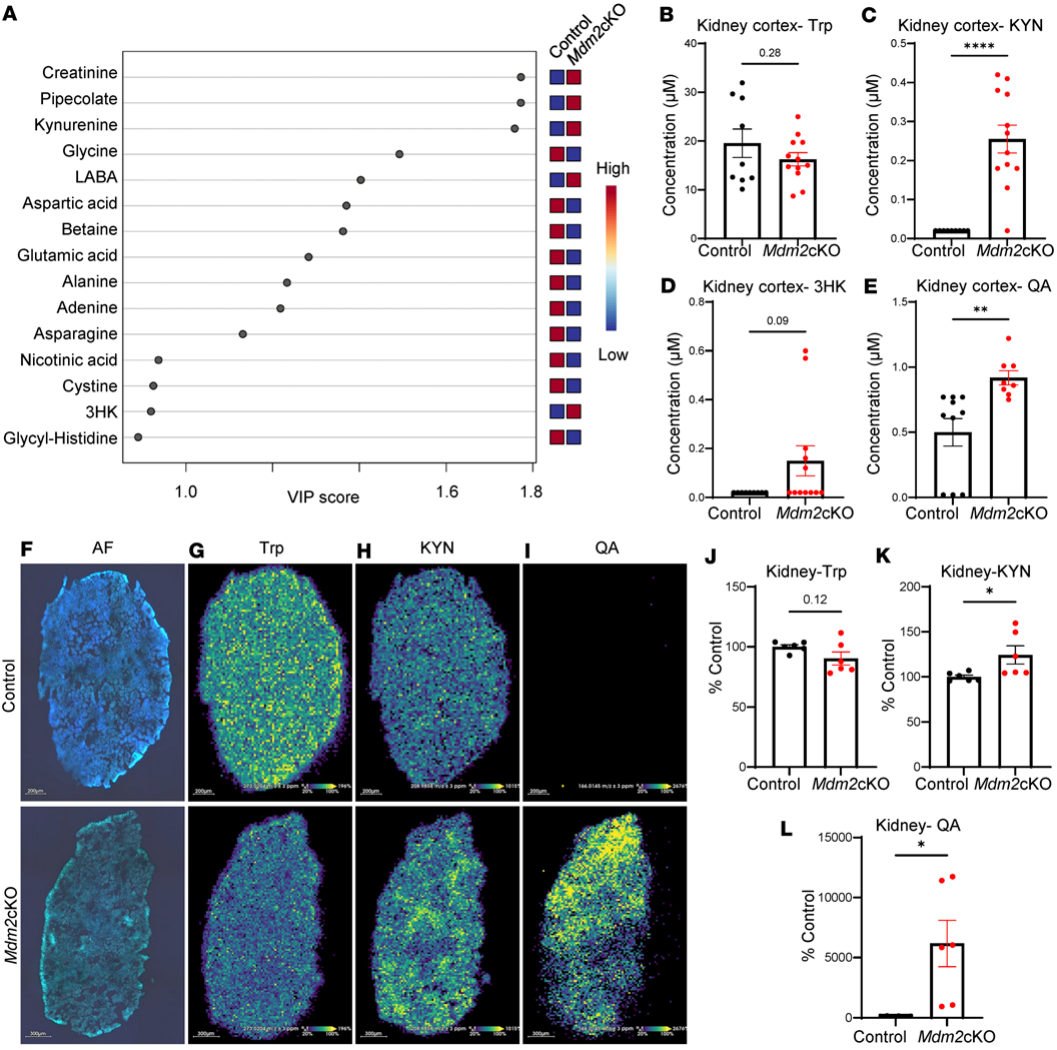
Trp metabolism is altered in the kidney during renal failure. Targeted metabolomics measured in kidney cortex tissue from *Mdm2*-cKO mice (*n* = 12) vs. control mice (*n* = 9) at day 6 of doxycycline administration, incorporating 32 metabolites of the amino acid panel. (**A**) Variable importance in projection (VIP) scores from partial least squares discriminant analysis (PLS-DA) of kidney metabolomics. Raw concentrations of (**B**) Trp, (**C**) KYN, (**D**) 3HK, and (**E**) QA. QA was measured with different mass spectrometry method (*Mdm2*-cKO *n* = 8; control *n* = 10). (**F**) Autofluorescence image of control (top; scale bar 200 μm) vs. *Mdm2*-cKO kidney section (bottom; scale bar 300 μm). Matrix-assisted laser desorption/ionization mass spectrometry imaging (MALDI-MSI) of kidney sections performed in duplicates from *Mdm2*-cKO vs. control (*n* = 3 per group) mice showing (**G**) Trp (*m/z* 273.0203), (**H**) KYN (*m/z* 208.9858), and (**I**) QA (*m/z* 166.0145), (**J**–**L**) with their respective pixel intensities’ semiquantifications displayed as percentage of control. Pixel intensity from blue (low) to yellow (high). Graphs display means ± SEM. *P < 0.05, **P < 0.01, and ****P < 0.0001 via 2-tailed *t* test.

Bulk metabolomics in the kidney cortex of *Mdm2*-cKO and control mice, using partial least squares discrimination analysis (PLS-DA), revealed separation between the groups. Among the top 15 metabolites ranked by variable importance in projection (VIP) scores, KYN was identified as a key contributor (Figure 3A and Supplemental Figure 5). Despite no changes in Trp concentration, the data revealed a significant increase in KYN and QA (Figure 3, B–E). Matrix-assisted laser desorption/ionization-mass spectrometry imaging (MALDI-MSI) verified the accumulation of KYN and QA in the kidney, as shown in Figure 3, F–L. Overlaying ion images with autofluorescence images revealed a diffuse QA distribution in both glomerular and nonglomerular regions of the *Mdm2*-cKO kidney sections, with greater prominence in areas of extensive tubular loss.

### Renal failure is associated with increased inflammatory profile and cell death in cortical brain regions.

**Figure 4:**
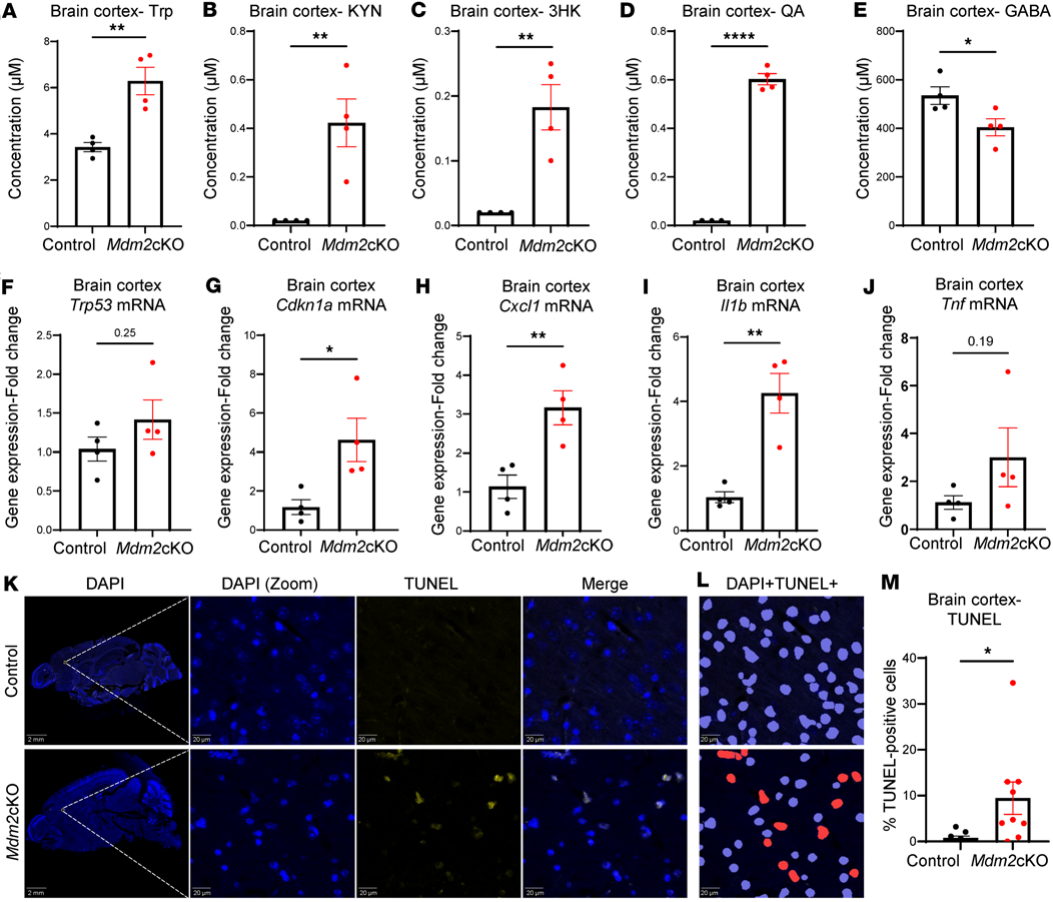
Brains of mice with rapid kidney failure exhibit accelerated KYN degradation and increased apoptosis. Targeted bulk metabolomics analysis in brain cortex tissue from *Mdm2*-cKO vs. control mice (*n* = 4 per group). Raw concentration of (**A**) Trp, (**B**) KYN, (**C**) 3HK, (**D**) QA, and (**E**) γ-aminobutyric acid (GABA). QA was measured with a different mass spectrometry method. RNA lysates were collected from the brain cortex of *Mdm2*-cKO vs. control mice (*n* = 4 per group). qPCR analysis (normalized to *Gapdh*) showing mRNA fold change in (**F**) *Trp53*, (**G**) *Cdkn1a*, (**H**) *Cxcl1*, (**I**) *Il1b*, and (**J**) tumor necrosis factor (*Tnf*). Terminal deoxynucleotidyl transferase–mediated dUTP nick end labeling standard (TUNEL) assay was conducted on brain sagittal FFPE sections obtained from *Mdm2*-cKO vs. control mice (*n* = 3 per group). (**K**) The panels on the left display sagittal brain sections of control (top) vs. *Mdm2*-cKO (bottom) mice; scale bar = 2 mm; white squares indicate a region of interest (ROI) magnified, as indicated by the dashed lines, showing DAPI^+^ (λex = 359 nm) in blue and TUNEL^+^ (λex = 594 nm) cells in yellow, and merged magnified images; scale bar = 20 μm. (**L**) QuPath cell detection imaging of nuclei (blue) and TUNEL-positive cells in red. (**M**) Graphical representation of percentage of TUNEL-positive cells in the *Mdm2*-cKO vs. control groups. Each data point represents an ROI in the brain cortex, 3 ROIs per sample. Graphs display means ± SEM. Two-tailed *t* tests: *P < 0.05, **P < 0.01, and ****P < 0.0001.

The majority of KYN within the brain comes from circulation via transport across the blood-brain barrier (21, 22). As inflammation triggers metabolism of KYN within the brain to generate oxidative and neurochemically active metabolites (22–24), we performed targeted bulk metabolomics analysis in the brain cortex of *Mdm2*-cKO versus control mice, focusing on Trp metabolites. There were elevated levels of Trp, KYN, 3HK, and QA in the brain cortex and a decrease in γ-aminobutyric acid (GABA) (Figure 4, A–E). Pathway analysis further highlighted Trp and glutamate metabolism in addition to ammonia recycling pathways, suggesting a mechanistic link to neuroinflammatory processes (Supplemental Figure 6 and Supplemental Table 2). Perturbed Trp metabolism was accompanied by changes in transcripts suggestive of a shift toward cell cycle arrest and inflammation as evidenced by significantly increased *Cdkn1a*, *Cxcl1*, and *Il1b* mRNA levels in the brain cortex of *Mdm2*-cKO mice (Figure 4, F–J). These alterations were accompanied by observable changes in cell death at the brain cortical area, underscoring the potential impact of QA on brain health. The percentage of TUNEL-DAPI–positive cells/total DAPI-positive cells was significantly increased in the *Mdm2*-cKO mice brain cortex compared with control (Figure 4, K–M).

It is noteworthy that the changes observed in the brain of *Mdm2*-cKO mice occurred while MDM2 levels in the brain were not decreased but slightly increased (Supplemental Figure 7), eliminating the possibility of off-target effects of *Mdm2* deletion.

### Spatial metabolomics reveals QA localization in ependymal cells during kidney failure.

**Figure 5:**
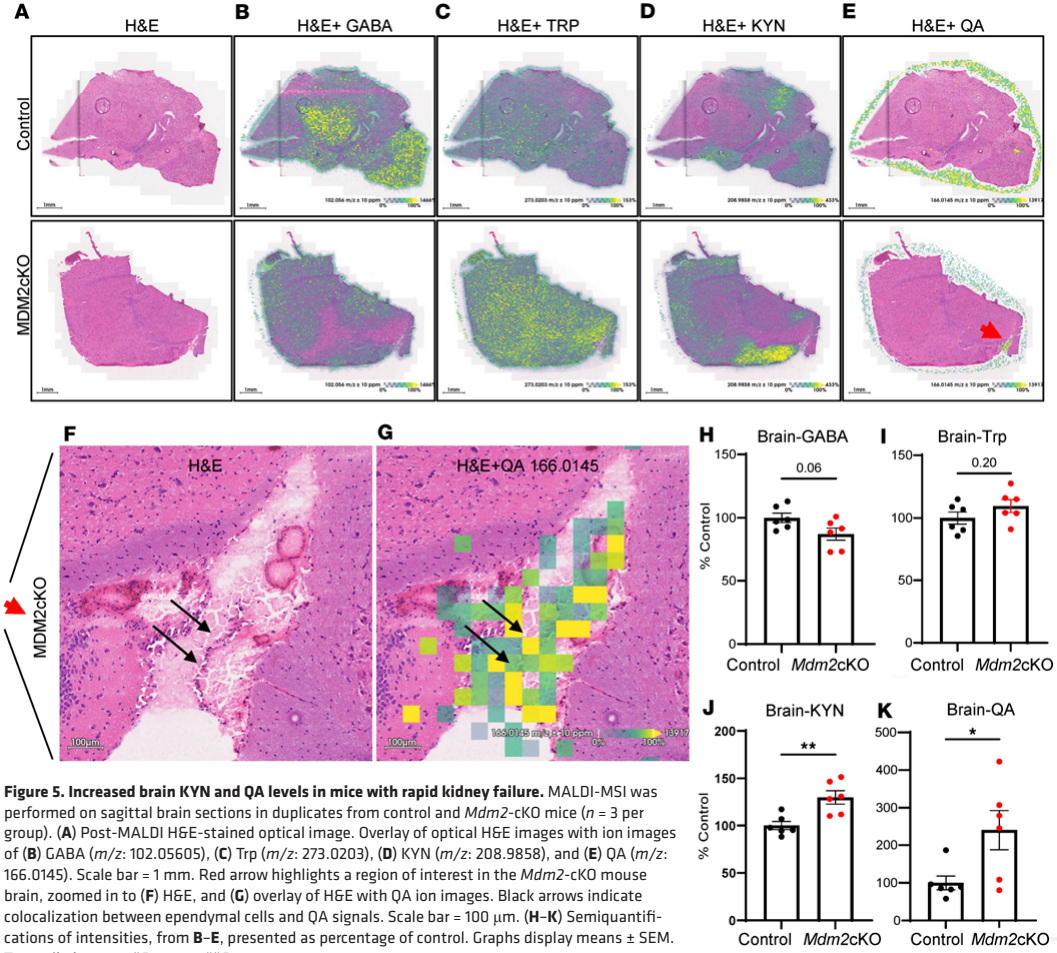
Increased brain KYN and QA levels in mice with rapid kidney failure. MALDI-MSI was performed on sagittal brain sections in duplicates from control and *Mdm2*-cKO mice (*n* = 3 per group). (**A**) Post-MALDI H&E-stained optical image. Overlay of optical H&E images with ion images of (**B**) GABA (*m/z*: 102.05605), (**C**) Trp (*m/z*: 273.0203), (**D**) KYN (*m/z*: 208.9858), and (**E**) QA (*m/z*: 166.0145). Scale bar = 1 mm. Red arrow highlights a region of interest in the *Mdm2*-cKO mouse brain, zoomed in to (**F**) H&E, and (**G**) overlay of H&E with QA ion images. Black arrows indicate colocalization between ependymal cells and QA signals. Scale bar = 100 μm. (**H**–**K**) Semiquantifications of intensities, from **B**–**E**, presented as percentage of control. Graphs display means ± SEM. Two-tailed *t* tests: *P < 0.05, **P < 0.01.

MALDI-MSI analysis was performed on sagittal freshly frozen brain sections in control and *Mdm2*-cKO mice with kidney failure. GABA was found in the caudate/striatum and thalamus of the control mouse brains and was slightly decreased within the *Mdm2*-cKO mice brain sections (Figure 5, A, B, and H). In contrast, Trp showed a trend of increase while there was a significant increase in KYN levels specifically across gray matter brain regions (Figure 5, C, D, I, and J). QA was not detectable in control brains by MALDI-MSI; however, the *Mdm2*-cKO mouse brains exhibited significantly increased QA intensity (Figure 5, E and K). Further analysis in one of the *Mdm2*-cKO mouse brains demonstrated specific localization of QA adjacent to or within ependymal cells, which line the cortical ventricle. This was verified by ion image overlay with optical images demonstrating QA colocalization to ependymal cells in post-MALDI H&E-stained tissue (Figure 5, F and G).

### Accelerated oxidative KYN degradation in ischemia/reperfusion model of severe AKI.

**Figure 6:**
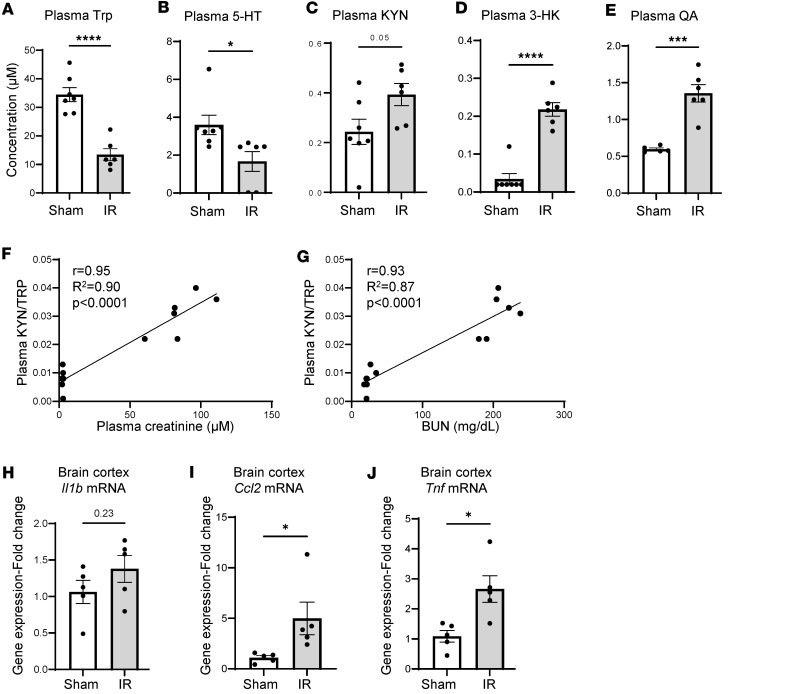
Ischemia-induced AKI mouse model exhibits increased inflammation and accelerated Trp degradation. Mice underwent bilateral kidney vasculature clamping for 35 minutes (IR) and were sacrificed 24 hours after reperfusion. Mass spectrometry was performed on plasma samples of IR mice (*n* = 6) vs. sham-operated mice as controls (*n* = 7). Raw concentrations of (**A**–**D**) Trp, serotonin (5-HT), KYN, 3HK, and (**E**) QA. QA was measured with a different mass spectrometry method (sham *n* = 5; IR *n* = 6). (**F** and **G**) Pearson correlations of the KYN-to-Trp ratio with plasma creatinine and BUN. (**H**–**J**) qPCR results of brain cortex RNA lysates (*n* = 5 per group), showing mRNA levels of *Il1b*, *Ccl2*, and *Tnf*, reported as fold-change relative to control. Graphs display means ± SEM. Two-tailed *t* test; *P < 0.05, ***P < 0.0 01, and ****P < 0.0001.

To verify that the changes in the brain inflammatory signaling milieu that we observed in the *Mdm2*-cKO mice could be due to common types of severe kidney tubular injury, we analyzed plasma samples of a mouse model of severe ischemic kidney injury. With 35 minutes of ischemia followed by 24 hours of reperfusion (IR), mice exhibited significant increases in BUN and plasma creatinine levels (Supplemental Figure 8). Interestingly, the IR mice exhibited a significant decrease in plasma Trp and 5-HT and a significant increase in 3HK and QA, similar to Trp metabolite patterns in plasma of *Mdm2*-cKO mice (Figure 6, A–E). Moreover, the KYN/Trp ratio strongly correlated to plasma creatinine and BUN levels (Figure 6, F and G). Brain analyses of cortical regions in the IR mice showed a trend toward increased mRNA levels of *Il1b*, accompanied by a significant increase in *Ccl2* and *Tnf* (Figure 6, H–J).

### Metabolic and inflammatory alterations in adenine-induced CKD mouse model.

**Figure 7:**
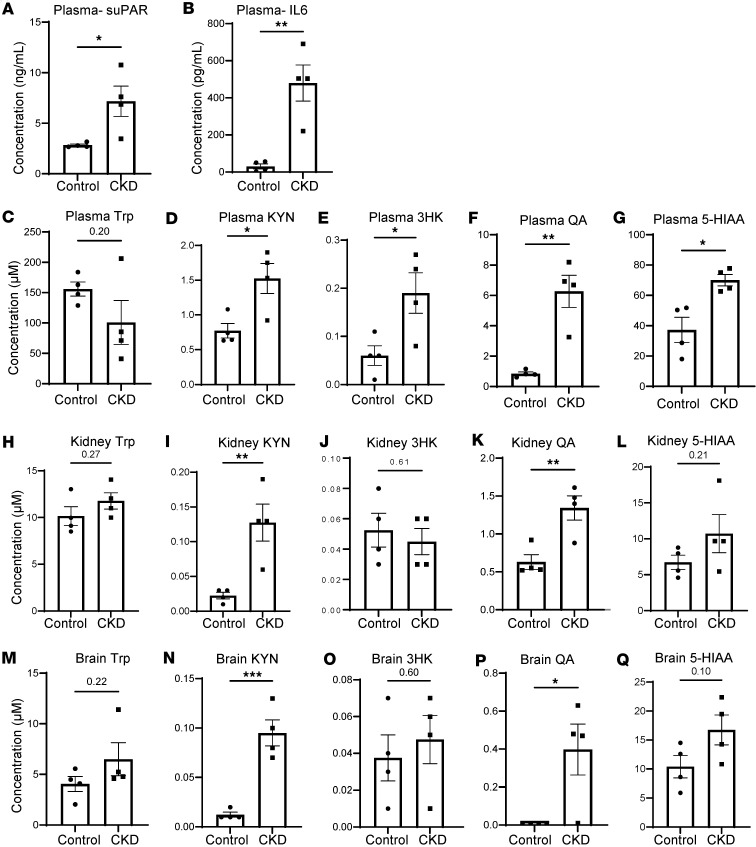
Ade-CKD alters systemic and tissue-specific Trp metabolism and inflammation. Mice were fed regular chow (control) or 0.2% adenine-enriched chow for 1 month (*n* = 4 per group). (**A** and **B**) Plasma suPAR and IL-6 levels measured by ELISA. Concentrations of Trp, KYN, 3HK, QA, and 5-HIAA in (**C**–**G**) plasma, kidney cortex (**H**–**L**), and brain cortex (**M**–**Q**). QA and 5-HIAA were measured using a different mass spectrometry method. Graphs display means ± SEM. Two-tailed *t* test; *P < 0.05, **P < 0.01, ***P < 0.001.

To further investigate the metabolic changes associated with kidney dysfunction in a more chronic context, we used the established adenine-induced CKD model (ade-CKD) (25, 26). The ade-CKD mice exhibited a significant increase in urinary albumin-to-creatinine ratio (ACR), plasma creatinine and BUN levels (Supplemental Figure 9), and plasma suPAR and IL-6, indicating kidney function loss and systemic inflammation (Figure 7, A and B).

Bulk metabolomics analysis revealed a significant increase in plasma KYN, 3HK, QA, and 5-HIAA (Figure 7, C–G), similar to the patterns observed in both the *Mdm2*-cKO and IR-induced AKI models. Within both the kidney and brain, we observed significant increases in KYN and QA (Figure 7, H–Q). These findings suggest that CKD impacts Trp metabolism both peripherally and locally in the kidney and brain. Correlation analysis revealed strong positive relationships between kidney dysfunction markers (BUN, ACR, creatinine) and systemic inflammation (IL-6, suPAR) in plasma (*r* > 0.9, *P* < 0.01). Plasma Trp was inversely correlated (*r* ~ –0.8, *P* < 0.05), and QA strongly correlated (*r* > 0.8, *P* < 0.01), with kidney dysfunction and inflammation, and the KYN-to-Trp ratio, reflecting enhanced IDO activity, showed a significant correlation with kidney dysfunction and inflammation (*r* > 0.9, *P* < 0.001) (Supplemental Figure 10).

In the kidney and the brain, QA levels were positively correlated with kidney dysfunction markers and systemic inflammation. The KYN-to-Trp ratio in both kidney and brain strongly correlated with markers of kidney dysfunction (*r* > 0.8, *P* < 0.01), highlighting systemic effects on brain metabolism (Supplemental Figures 11 and 12).

**Figure 8:**
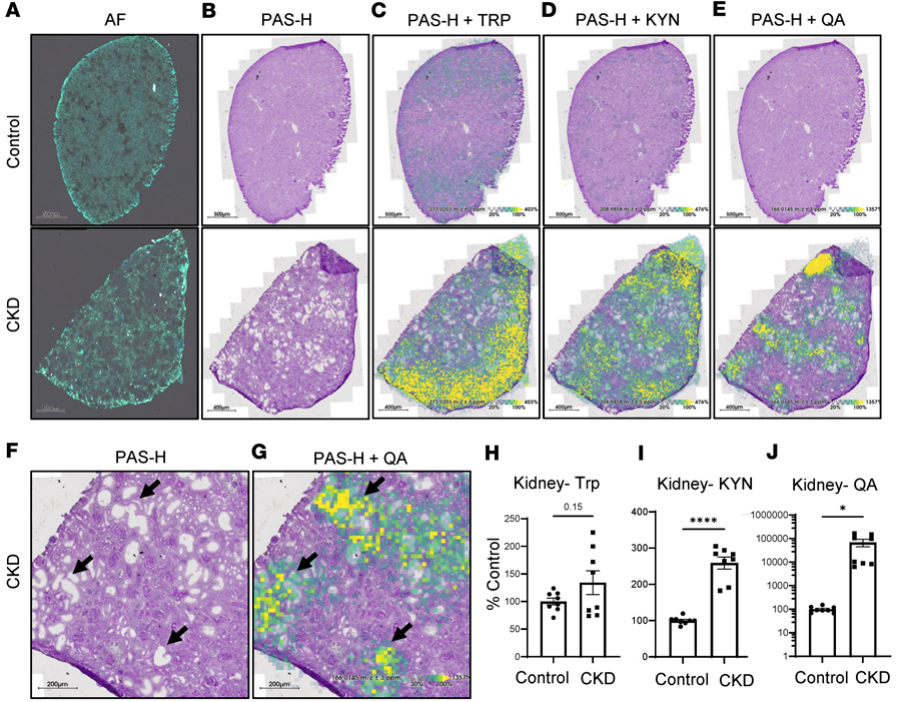
Spatial metabolomics reveals colocalization of QA with tubular injury in ade-CKD mice. Serial kidney sections from control vs. CKD mice (*n* = 4 per group) were analyzed in duplicates using MALDI-MSI. (**A**) Autofluorescence (AF) image of kidney sections from control (top, scale bar = 500 μm) and CKD (bottom, scale bar = 400 μm) (pre-MALDI). (**B**) Periodic acid–Schiff hematoxylin (PAS-H) staining of serial sections. (**C**–**E**) PAS-H staining overlaid with ion images for Trp (*m/z*: 273.0203), KYN (*m/z*: 208.9858), and QA (*m/z*: 166.0145), respectively. (**F** and **G**) Zoomed-in CKD images from **C** and **E**, with black arrows indicating regions of colocalization between tubular dilation and QA signals; scale bar = 200 μm. (**H**–**J**) Semiquantification of ion intensities displayed as a percentage of control. Graphs display means ± SEM. Two-tailed *t* test; *P < 0.05, and ****P < 0.0001.

Interestingly, MALDI-MSI analysis revealed a notable accumulation of QA specifically within kidney regions associated with injury (Figure 8). Multimodal spatial analysis verified this pattern, highlighting QA localization in tubular areas exhibiting dilation and epithelial cell loss. These findings align with observations in the *Mdm2*-cKO model, where extensive tubular cell loss was coupled with consistent QA elevation across kidney sections.

To test the effect of QA on kidney tubules, we treated human kidney (HK2) cells with low doses of QA (2.5 μM and 5 μM) for 24 hours, concentrations that reflect levels of QA found in the plasma of CKD mouse models. We observed a concentration-dependent increase in the protein levels of fibronectin (FN1) and collagen, type I, α2 (COL1A2), fibrosis markers (Supplemental Figure 13), suggesting that QA accumulation may contribute to fibrotic changes in the kidney tubules.

Overall, these results demonstrate that both peripheral and tissue alterations in Trp metabolism are associated with CKD, with marked accumulation of neurotoxic metabolites such as KYN and QA, potentially contributing to neurological complications of kidney disease and exacerbating kidney dysfunction.

### Advanced kidney disease in humans is associated with increased Trp degradation via the KP.

**Figure 9:**
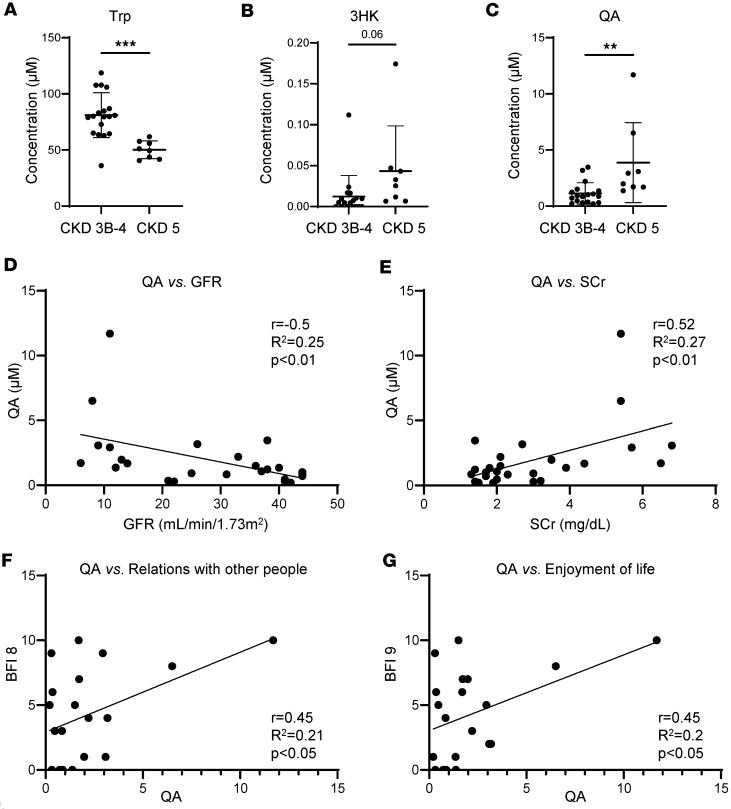
Enhanced Trp degradation and inverse correlation of QA and kidney function in patients with CKD. Plasma Trp metabolites were quantified using liquid chromatography-mass spectrometry (LC-MS) in patients with CKD not on dialysis (*n* = 8 for CKD stage 5; *n* = 18 for CKD stage 3b and 4). (**A**–**C**) Plasma concentrations of Trp, 3HK, and QA. (**D**) Pearson correlation between QA and estimated glomerular filtration rate (eGFR). (**E**) Pearson correlation between QA and serum creatinine. (**F**) Pearson correlation between plasma QA concentration and fatigue interference in relations with other people. (**G**) Pearson correlation between plasma QA concentration and fatigue interference in enjoyment of life. Graphs display means ± SD. Two-tailed *t* test: **P < 0.01, and ***P < 0.001. BFI, Brief Fatigue Inventory.

In a cohort of CKD patients with a primary etiology of type 2 diabetes or hypertension (Supplemental Tables 3–5), we observed that levels of plasma Trp were decreased and levels of 3HK and QA were elevated in patients with CKD stage 5 not on dialysis (3HK mean = 0.04; SD = 0.06; *P* = 0.06 and QA mean = 3.87; SD = 3.56; *P* < 0.01), compared with CKD stages 3b and 4 (3HK mean = 0.01; SD = 0.03 and QA mean = 1.13; SD = 0.95) (Figure 9, A–C). These findings, based on a sample size of 8 patients with CKD stage 5 and 18 with stages 3b and 4, indicated a potential rise in neurotoxins associated with the progression of kidney disease severity, similar to the mouse models.

The physiological reference range for plasma 3HK in healthy individuals is typically less than 0.13 μmol/L (27). In another study, plasma 3HK levels ranged from 0.015 to 1.5 μM, with an average of 0.0258 μM in 10 healthy volunteers (28). Plasma QA levels in healthy individuals ranged from 0.025 to 1 μM, with an average of 0.267 μM (28).

In our cohort, the elevated levels of 3HK and QA, particularly in CKD stage 5, may suggest an abnormal degradation of Trp in severe kidney disease. Plasma QA was inversely correlated with the estimated glomerular filtration rate (eGFR) and directly correlated with serum creatinine levels in CKD (Figure 9, D and E). This suggests that as kidney function declines in humans, there is a concurrent accumulation of QA.

Examination of the relationship of psychosocial measures and Trp metabolites revealed that higher QA levels were associated with greater fatigue interference in aspects of quality of life, such as relationships with others (*r* = 0.45, *P* < 0.05) and enjoyment of life (*r* = 0.45, *P* < 0.05) (Figure 9, F and G). Correlation analysis did not reveal association of fatigue with other Trp metabolites, and we could not find any association with other Brief Fatigue Inventory (BFI) items (data not shown). Moreover, individual BFI item scores did not differ between patients with CKD stages 3b-4 and 5. These findings suggest that the metabolic disturbance in CKD is particularly associated with QA and that QA may have a profound impact on the quality of life.

## Discussion

In 2 mouse models of severe kidney dysfunction and in a mouse model of CKD, as well as in patients with advanced CKD, we observed similar accumulation of downstream bioactive Trp metabolites in the blood. We report that *Mdm2*-cKO mice exhibited kidney and systematic inflammation consequent to massive kidney tubular cell death. Similarly, the ade-CKD mice also exhibited increased systemic inflammation correlating with kidney dysfunction. The enhanced 5-HT degradation and the accelerated KYN degradation toward the oxidative KP are likely driven by the inflammatory response of the damaged kidney at the local and systemic levels. Furthermore, QA accumulation in the CKD model mirrors the elevated circulating levels of QA observed in human samples from patients with advanced CKD, underscoring the translational relevance of our findings. It is noteworthy that targeting the KP has been shown to improve kidney injury in KMO-null mice (29). Inflammation leading to Trp oxidative degradation is widely supported by previous studies (30–32). This shift has profound implications as it can lead to the production of various neurotoxic and immunoregulatory metabolites. It is notable that the accumulation of 3HK and QA correlates with risk of developing cognitive impairment and dementia (33, 34) and several neurological disorders wherein altered inflammatory signaling is a key component (24, 32, 35, 36). Moreover, KMO inhibition peripherally increased kynurenic acid levels, reduced glutamate, and showed potential in preventing neurodegeneration in Alzheimer’s and Huntington’s disease mouse models (37).

In addition, we observed elevated plasma suPAR levels in both the kidney tubular cell loss (*Mdm2*-cKO) and the ade-CKD experimental models versus control mice. These findings are consistent with prior studies, such as Hayek et al. (20, 38), which demonstrated the role of suPAR in AKI and inflammation and linked suPAR to eGFR decline and CKD progression, and other studies (17–19, 39), which identified association of suPAR in the serum and CSF of patients with neurological diseases.

### Role of tubular injury in systemic inflammation and KYN degradation.

Combining data from the *Mdm2*-cKO mouse model of tubular cell death and the IR model validates a causative role for tubular injury leading to systemic inflammation and KYN degradation. Previous studies have demonstrated a dualistic nature of tubular cells, which can be both susceptible to systemic inflammation and actively contribute to the generation of inflammatory mediators (16). These processes can affect the progression of kidney injury and its impact on distant organs. Using spatial metabolomics multimodal analysis of ade-CKD kidneys, we detected QA accumulation specifically in kidney regions associated with tubular injury. This pattern was consistent with *Mdm2*-cKO kidney analysis, suggesting that tubular loss in both acute and chronic kidney injury settings involving inflammation leads to QA accumulation, which may exacerbate and prolong kidney damage by inducing oxidative stress contributing to fibrosis (40, 41). A study in patients with end-stage renal disease (42) suggested that elevated plasma QA levels are associated with increased oxidative stress and inflammation. Our in vitro studies showed that low doses of QA increased FN1 and COL1A2 protein levels in HK2 cells, indicating a potential role for QA in prolonging tubular injury. These findings align with research by Clark et al. (43), which demonstrated that increasing quinolinate phosphoribosyltransferase (QPRT), an enzyme that converts QA into nicotinamide dinucleotide (NAD^+^), mitigated QA’s toxic effects and improved kidney function. Thus, while QA accumulation is likely secondary to tubular injury, it may contribute to disease progression by exacerbating oxidative stress and promoting fibrosis.

### Trp metabolism via the KP in the brain is associated with increased QA, inflammation, and cell death.

Pathway enrichment analysis from the targeted bulk metabolomics of brain cortical lysates in *Mdm2*-cKO mice highlighted Trp and glutamate pathways among the top 5 altered pathways. The interaction between Trp metabolism through the oxidative KP and glutamate metabolism in the brain presents an intriguing avenue for exploration. Oxidative KP is driven by inflammation in the brain (44). Our data in both mouse models and prior research document altered brain inflammatory profiles under kidney injury (45). Interestingly, QA is a bioactive KYN oxidative by-product, typically found in very low nanomolar levels (<100 nM) in the human brain and CSF (46, 47). However, QA can reach toxic levels in response to inflammation. Similarly, our quantitative bulk metabolomics analysis demonstrated undetectable QA in the control brains versus an average of 0.6 μM QA in the *Mdm2*-cKO brain cortex. QA is frequently referred to as an excitotoxic or neurotoxic brain metabolite and has been associated with the development of various neurological diseases in humans (23). QA’s role as a potent neurotoxin is well documented. QA interacts with NMDA receptors, competing with glutamate, and contributes to excitotoxicity and neuronal damage (23, 48–51). Moreover, our finding of decreased GABA levels in the brain cortex is consistent with other studies showing GABA as a suppressor of inflammation and excitotoxic damage (52, 53), and its decrease in the brain is associated with various neurological disorders (54, 55).

These findings align with a recent study (56) showing that uremic plasma increases microglial activation and IL-1β signaling, contributing to neuroinflammation. This study further demonstrated that plasma from patients with CKD disrupts blood-brain barrier integrity, leading to increased permeability and neuronal dysfunction. This is consistent with other reports that implicate uremic toxins in blood-brain barrier disruption, behavioral changes, and neuronal impairment (12, 57). Given our data showing significant alterations in KYN metabolism and excitotoxic metabolites in the *Mdm2*-cKO, IR, and ade-CKD mice brains, it is plausible that kidney dysfunction–driven systemic inflammation exacerbates blood-brain barrier dysfunction and promotes neuroinflammatory changes, further contributing to neuronal impairment.

Our spatial metabolomics approach overlaying MALDI-MSI ion images to post-MALDI H&E-stained brain tissue revealed an interesting pattern wherein QA colocalized with ependymal cells in one of the *Mdm2*-cKO brains. Ependymal cells are glial cells that line the cerebral ventricles and secrete and circulate CSF. Interestingly, CSF QA levels have been correlated with neurological disorders associated with conditions such as HIV infection, traumatic brain disorder, and hepatic encephalopathy (58–63). Moreover, the QA catabolic enzyme QPRT was observed via immunohistochemistry in ependymal cells of the cerebral ventricles (64). Cumulatively, our data and published studies indicate that QA is closely implicated in inflammation in the brain and excitotoxicity and that ependymal cells may play a role in the metabolism or regulation of quinolinic acid in the brain. However, the functional significance of the localization of QA to ependymal cells has not yet been investigated.

### Inflammation, favoring oxidative Trp catabolism, could lead to fatigue.

A notable percentage of patients with CKD not on kidney replacement therapy report fatigue (65, 66). In general, patients with CKD have a worse quality of life (65, 67). However, the underlying mechanisms are poorly understood. We and others have previously demonstrated an inverse association between circulating Trp levels and stages of CKD (30, 68). KYN, kynurenic acid, and QA were positively and robustly correlated with the severity of kidney disease due to higher enzymatic activity induced mainly by inflammation.

In the present study, we consistently found that patients with CKD stage 5, compared with stages 3b/4, had low circulating Trp levels coupled with high levels of KP pathway metabolites, indicating enhanced IDO activity. Here, we also observed a strong correlation specifically between plasma QA and scores of fatigue interference with certain daily activities, notably relationships with people and enjoyment of life. To our knowledge, these are the first data linking QA specifically to psychosocial changes in patients with CKD.

Our data are consistent with several reports that showed correlation of QA with fatigue in patients with chronic fatigue syndrome, fibromyalgia, and systemic lupus erythematosus (69, 70). Reducing KYN in postmenopausal breast cancer survivors has been demonstrated to correlate with reduced fatigue (71). Our research holds particular relevance for neurologic dysfunction associated with progressive CKD and AKI. Ongoing research into the kidney/brain axis via metabolic alterations arising from the kidney offers exciting prospects for targeted treatments.

### Limitations.

While our study offers insights into the connections between kidney dysfunction, Trp metabolism, and brain inflammation within a spatial context, there are limitations that should be considered when interpreting the findings. First, our findings indicate that in the *Mdm2*-cKO model of kidney failure, there are no observed effects consistent with diffuse multiorgan failure, thus supporting our hypothesis of a kidney/brain axis. However, we acknowledge that there are likely potential effects of metabolism involving multiple organs that are triggered by kidney dysfunction and could also contribute to brain dysfunction.

Second, we consistently observed plasma Trp was reduced but that brain levels were increased. This suggests that despite systemic depletion, the brain may prioritize Trp uptake for 5-HT synthesis, crucial for neurotransmitter balance and neuroprotection. While the underlying mechanism remains speculative, our data are consistent with our previous findings in mice showing peripheral immune challenge with lipopolysaccharide results in a reduction in circulating Trp levels with simultaneous increase in brain Trp levels (72). A similar disconnection between peripheral and brain (CSF) Trp levels was noted in patients undergoing IFN-α immunotherapy (73). Here, we suggest that kidney injury–induced inflammation could disrupt the blood-brain barrier, altering transporter activity or shifting metabolic pathways to favor 5-HT production in the brain. These findings highlight the complex relationship between systemic injury and brain homeostasis. Further studies, including labeled isotope tracking, are needed to clarify the mechanisms driving this discrepancy.

Last, our clinical study included a relatively small sample size, with 18 patients in CKD stages 3b-4 and 8 patients in CKD stage 5. Measurements of these biomarkers and their associations with fatigue, depression, and cognitive decline will need to be carried out in larger cohorts in future studies.

Overall, our study provided strong evidence, with data from 3 separate mouse models of AKI and CKD as well as a small patient cohort study, to demonstrate that systemic inflammation resulting from impaired kidney function leads to QA accumulation and may exacerbate kidney damage and contribute to a toxic environment in the brain. While most of our data are contextual, they highlight important mechanisms at play, with translational significance for understanding the metabolic connections between acute and chronic kidney disease and brain alterations.

## Methods

### Sex as a biological variable.

In the human studies, both male and female samples were included; however, sex was not analyzed as variable, as there were few patients to be analyzed separately. In the *Mdm2*-cKO studies, both male and female mice were used. However, experiments were not designed to include equal numbers of each sex, and sex-based differences were not analyzed. For the CKD and IR mouse studies, only male mice were used, as females are known to have greater tolerance to IR and slower kidney disease progression (74, 75). Thus, it remains unclear whether these findings are fully applicable to females.

### Mdm2-CKO mice.

*Pax8*-rtTAcre *Mdm2*^fl/fl^ or *Mdm2*^fl/fl^ control 3- to 5-month-old mice, maintained on a C57BL/6J background, were treated with doxycycline 2 mg/mL in 5% sucrose drinking water (6–20 days) to induce deletion of *Mdm2* in paired box 8–positive (*Pax8*-positive) cells (13, 14, 76). Mice were monitored daily during doxycycline administration. Blood was collected via submandibular vein for metabolomics analysis (capillary electrophoresis–mass spectrometry [CE-MS]; ZipChip) or complete blood count blood analysis (VetScan-HM5-Hematology Analyzer). Primers for mouse genotyping are detailed in Supplemental Table 6. *Pax8*-rtTAcre mice were provided as a gift from Karen Block, The University of Texas Health Science Center at San Antonio, and the *Mdm2*^fl/fl^ mice were obtained from MD Anderson Cancer Center (77).

### IR injury in mice.

Ten- to 12-week-old male C57BL/6J mice (The Jackson Laboratory; 000664) were anesthetized by isoflurane inhalation (3% in oxygen), and body temperature was maintained at 36°C–37°C. Bilateral renal IR was conducted as previously described (78) with some modifications. Briefly, the renal pedicles were clamped for 35 minutes with small nontraumatic vascular aneurysm clips (Roboz Surgical Instruments). Subcutaneous saline injection of 0.5 mL was administered after performing 2-layered incisions. The same surgical procedures, except for pedicle clamping, were applied on sham-operated mice.

### Ade-CKD in mice.

C57BL/6J mice, 3–5 months old, were fed with a standard chow diet (*n* = 4) or a 0.2% adenine-supplemented diet (Inotiv TD.230441) for a duration of 1 month (25, 26). At the end of the treatment, tissue samples were snap-frozen or slowly frozen using isopentane and stored at –80°C until analysis.

BUN was measured using the assay kit (Arbor Assays, K024-H) according to manufacturer’s instructions.

### Targeted bulk metabolomics.

Amino acid and Trp metabolites were measured from plasma, frozen kidney cortex, or brain cortex lysates (10 μL) using ZipChip (908 Devices) coupled with MS or by bulk LC-MS analysis (15, 79, 80). The plasma samples were treated in the same manner as the tissue lysates. A microfluidic chip that integrates CE with nano-electrospray ionization through a ZipChip interface separates metabolites. LC-MS analyses were performed on the Thermo Q Exactive HF-X Orbitrap mass spectrometer (Thermo Fisher Scientific) interfaced with heated electrospray ionization source and coupled with Thermo Fisher Scientific Vanquish HPLC system. An aliquot of 5 μL of the sample was injected into the instrument using an autosampler. The chromatographic separation took place on an Agilent ZORBAX HILIC PLUS column with 3.5 μm particle size and with the dimensions 2.1 × 100 mm with a phase composition of 10 mM ammonium formate, 0.05% formic acid, in Millipore water (component A; MilliporeSigma), and 0.05% formic acid in acetonitrile (component B) at 0.3 mL/min flow rate (81).

QA and 5-HIAA were analyzed with the XBridge Peptide BEH C18 column with 2.5 μm particle size and with the dimensions 2.1 × 100 mm with a phase composition of 10 mM ammonium acetate, and 0.05% acetic acid in 100% acetonitrile (component B) at 0.3 mL/min flow rate was used. Data acquisition and processing were carried out using Thermo Fisher Scientific’s Xcalibur Quant Browser software.

### MALDI-MSI.

Frozen brain or kidney tissue sections from mice were subjected to MALDI-MSI in negative ion mode using an Orbitrap mass spectrometer operated at a resolution of 120,000 at *m/z* 200. Cryosectioned tissue samples were coated with a matrix compound, and mass spectra were acquired in imaging mode to generate high-resolution ion images, with details described before (82). The annotations were processed using CoreMetabolome — v3 and HMDBI on METASPACE. Images were extracted on SCiLS Lab software followed by total ion current normalization to account for variations in ion intensity across the tissue sections.

### RNA extraction and qPCR.

Total RNA was extracted using QIAGEN RNeasy Mini Kit (catalog 74104). Briefly, 500 μL lysate was mixed with 500 μL of 100% ethanol and applied to RNeasy Mini spin column. After washing steps, RNA was eluted in 30–50 μL of RNase-free water. The RNA concentration and purity were determined using NanoDrop spectrophotometer. cDNA synthesis was performed using Thermo Fisher Scientific RevertAid Reverse Transcription Kit (catalog 4374966) with a total of 1 μg of RNA for each sample reaction. qPCR master mix was prepared by mixing 5 μL SYBR Green PCR Master Mix (Thermo Fisher Scientific, catalog A25780) with 0.5 μL primer mix (forward and reverse 10 μM) and nuclease-free water to reach a final reaction volume of 11 μL per reaction. The primer sequences are listed (Supplemental Table 7).

### Protein analysis.

Protein extraction from kidney cortex was performed using RIPA buffer (Abcam, ab156034) with phosphatase and protease inhibitor cocktail (Thermo Fisher Scientific 78440). Total protein concentration was quantified using the BCA protein assay kit (Thermo Fisher Scientific, 23235).

### Western blot.

Equal amounts of kidney cortex protein lysates were used for Western blotting. Primary antibodies include p53 (Santa Cruz Biotechnology, sc-126), NOX2 (Thermo Fisher Scientific, MA5-35348), MDM2 (Thermo Fisher Scientific, MA1-24643), p21 (Abcam, ab109199), and actin (C-2) (Santa Cruz Biotechnology, sc-8432). Quantification of protein bands was performed using ImageJ (NIH) (83).

### ELISA protein quantifications.

Quantitative protein levels in plasma and tissue lysates were assessed using ELISA kits following manufacturer’s protocols: mouse uPAR DuoSet ELISA (R&D Systems, Bio-Techne, DY531), mouse CXCL1/KC Quantikine ELISA Kit (R&D Systems, Bio-Techne, MKC00B-1), mouse IL-6 Quantikine ELISA Kit (R&D Systems, Bio-Techne, M6000B-1), and mouse IL-1β/IL-1F2 Quantikine ELISA Kit (R&D Systems, Bio-Techne, MLB00C-1).

### TUNEL assay.

TUNEL assay was performed using the Elabscience (E-CK-A322) kit. Brain tissue samples were collected and stored in 70% ethanol at 4°C after 24-hour fixation in 10% formalin, followed by paraffin embedding and sagittal sectioning. Positive and negative control measures were employed. The TUNEL assay results were analyzed using QuPath software (84). Three regions of interest were selected in the cortex of every sample at similar locations from control (*n* = 3) versus *Mdm2*-cKO (*n* = 3). The fluorescent signal was captured using Zeiss Axioscan 7 microscope equipped with a 10×/0.45 Plan Apochromat objective, with excitation wavelengths λex = 359 nm (DAPI) and λex = 594 nm (TUNEL).

### Study population.

In this observational study, we enrolled adults with CKD treated at an adult outpatient nephrology clinic with the following eligibility criteria: (a) clinical diagnosis of CKD stages 3b, 4, or 5; (b) primary etiology of CKD is either type 2 diabetes and or hypertension; and (c) on standard management for diabetes, hypertension, and associated comorbidities including anemia as per the recommended guidelines (85). The study was approved by the local Institutional Review Board (IRB), and all participants provided written informed consent prior to the study procedures.

### Data collection.

During a routine clinic visit, each participant completed the Brief Fatigue Inventory to report fatigue. Random blood was collected from each consented participant in a serum separator tube for serum and an anticoagulant-containing vacutainer for plasma. Each participant also provided random spot urine, which was measured for ALB and creatinine as per standard methodologies. Relevant sociodemographic (age, sex, height, and weight), clinical (medical history and concomitant medications), and laboratory data (e.g., glycated hemoglobin, blood hemoglobin) were reviewed and abstracted from the electronic health record. Serum creatinine was measured using a kinetic rate Jaffé method. The eGFR was calculated using serum creatinine, age, and sex based on the Chronic Kidney Disease Epidemiology Collaboration equation (86). Supplemental Table 8 shows the stages of CKD based on eGFR categories.

### Trp metabolites.

Free Trp and selective metabolites in the KP were measured in plasma by LC-MS as previously reported (44). Briefly, 50 μL plasma was diluted with 5× 0.2% acetic acid. Stable isotope–labeled standards, 2-picolinic-d4 acid, 2,3-pyridinedicarboxylic acid-d3, l-Trp-13C11,15N2, and KYN, were added at the time of extraction as internal standards for absolute quantification. The diluted samples were vortexed and transferred to 0.5 mL MilliporeSigma Amicon Ultra filter (3 kDa). The filter tubes were centrifuged at 13,500*g* for 60 minutes at 4°C, and the extracts were transferred to glass vials for LC-MS analyses. High-performance liquid chromatography (HPLC)/electrospray ionization MS analyses were conducted on a Thermo Fisher Scientific Q Exactive mass spectrometer with online separation by a Thermo Fisher Scientific/Dionex Ultimate 3000 HPLC. HPLC conditions were as follows: column, YMC-Pack ODS-AQ, 3 μm, 2 × 100 mm (YMC); mobile phase A, 0.5% formic acid in water; mobile phase B, 1% formic acid in acetonitrile; flow rate, 200 μL/min; gradient, 1% B to 30% B for 5 minutes and held at 70% B for 5 minutes to clean the column. The MS analyses were conducted using full MS scan (70,000 resolution) with positive ion detection. Standard curves were generated for all targeted KYN compounds using appropriate stable isotope–labeled internal standards and native compounds. Quantitative results were obtained by reference of the experimental peak area ratios to the standard curves.

### Brief Fatigue Inventory.

The Brief Fatigue Inventory is a 9-item questionnaire that measures fatigue during the past 24 hours on a 0–10 Likert scale with higher scores representing worse fatigue (87). The first 3 Brief Fatigue Inventory items measure fatigue severity with score ranging from 0 (no fatigue) to 10 (fatigue as bad as you can imagine). The remaining 6 Brief Fatigue Inventory items assess fatigue interference in relation to patients’ general activity, mood, walking ability, normal work (both indoor and outdoor), relations with other people, and enjoyment of life. These fatigue interference items represent the pervasive impact of fatigue on daily life activities — one of the most important and prioritized outcomes in patients with CKD (88). Fatigue interference items are measured on a 0–10 numerical rating scale, with 0 being “does not interfere” and 10 being “completely interferes.”

### Blinded research.

Metabolomics measurements (bulk and spatial) were conducted blindly by research technician experts without access to samples’ group identification. Multiorgan histopathological assessments were performed in a blinded manner by an expert pathologist.

### Statistics.

For human studies, descriptive data are presented as mean ± SD, and all comparisons are 2-tailed unpaired *t* test. We performed Pearson correlation coefficient test to measure the association between 2 variables. For mouse studies, 2-tailed unpaired *t* test for comparison between groups was employed, and data were presented as mean ± SEM. Moreover, Pearson correlation test was performed. All analyses were performed on GraphPad Prism 8 with *P* < 0.05 reported as significant. MetaboAnalyst 5.0 (89) was used for metabolic data analysis, principal component analysis, and PLS-DA to scrutinize metabolite differences between groups. For metabolic enrichment analysis, SMPDB was employed to recognize metabolic pathways significance reported with FDR-adjusted *P* < 0.05.

### Study approval.

For human studies, all study procedures including biospecimen collections were performed after obtaining written consent to the IRB-approved study protocol from each participant (The University of Texas Health Science Center at San Antonio IRB approval 20140210HU). Mouse studies were carried out after institutional animal care and use committee approval from The University of Texas Health Science Center at San Antonio.

### Data availability.

All the data necessary to support the conclusions presented in this manuscript are included within the figures, tables, or supplemental material unless clearly stated otherwise. Raw data are available in the Supporting Data Values XLS file.

Further information can be found in Supplemental Methods.

## Author contributions

AS and KS conceptualized and led the research. AS designed and conducted experiments; acquired, analyzed, interpreted, and presented the data; and wrote the manuscript. SD conducted the clinical research. IT performed MALDI-MSI analysis. HJL and RM conducted experiments (CKD in mice). FD and RM performed protein analysis. NR performed MS. JT and MM conducted experiments (IR in mice). SM and CF contributed to data acquisition. ET and PS contributed to sample and data acquisition. Authors provided expert guidance and data assessment as follows: LH (MS data quality control), GZ (metabolomics), SD (clinical research, CKD, and Trp metabolism), MAV (multiorgan histopathological assessment), JCO (inflammation, Trp metabolism, neuroscience), BF (data analysis, neuroscience), SCH (brain histology, inflammation), KFB (brain histology and interpretation), JDL (metabolism, neuroscience). All authors reviewed, provided edits to, and approved the submission of the manuscript. KS provided oversight and scientific and grant acquisition support for all the work.

## Supplementary Material

Supplemental data

Unedited blot and gel images

Supporting data values

## Version 1 02/13/2025

In-Press Preview

## Version 2 03/24/2025

Electronic publication
