# Supplementary material for: Quinolinic acid potentially links kidney injury to brain toxicity
Source: JCI Insight. 2025 Feb 13;10(6):e180229. doi: 10.1172/jci.insight.180229 (PMC11949017; doi:10.1172/jci.insight.180229)
Supplement: Supplemental data [file jciinsight-10-180229-s069.pdf]

Supplemental File

Title: Quinolinic acid potentially links kidney injury to brain toxicity

Saliba et al.

Content:

Supplemental Figures

Supplemental Tables

Supplemental Methods

Supplemental References

Supplemental Figure 1: Characterization of the *Mdm2* conditional knock-out rapid kidney failure mouse model.

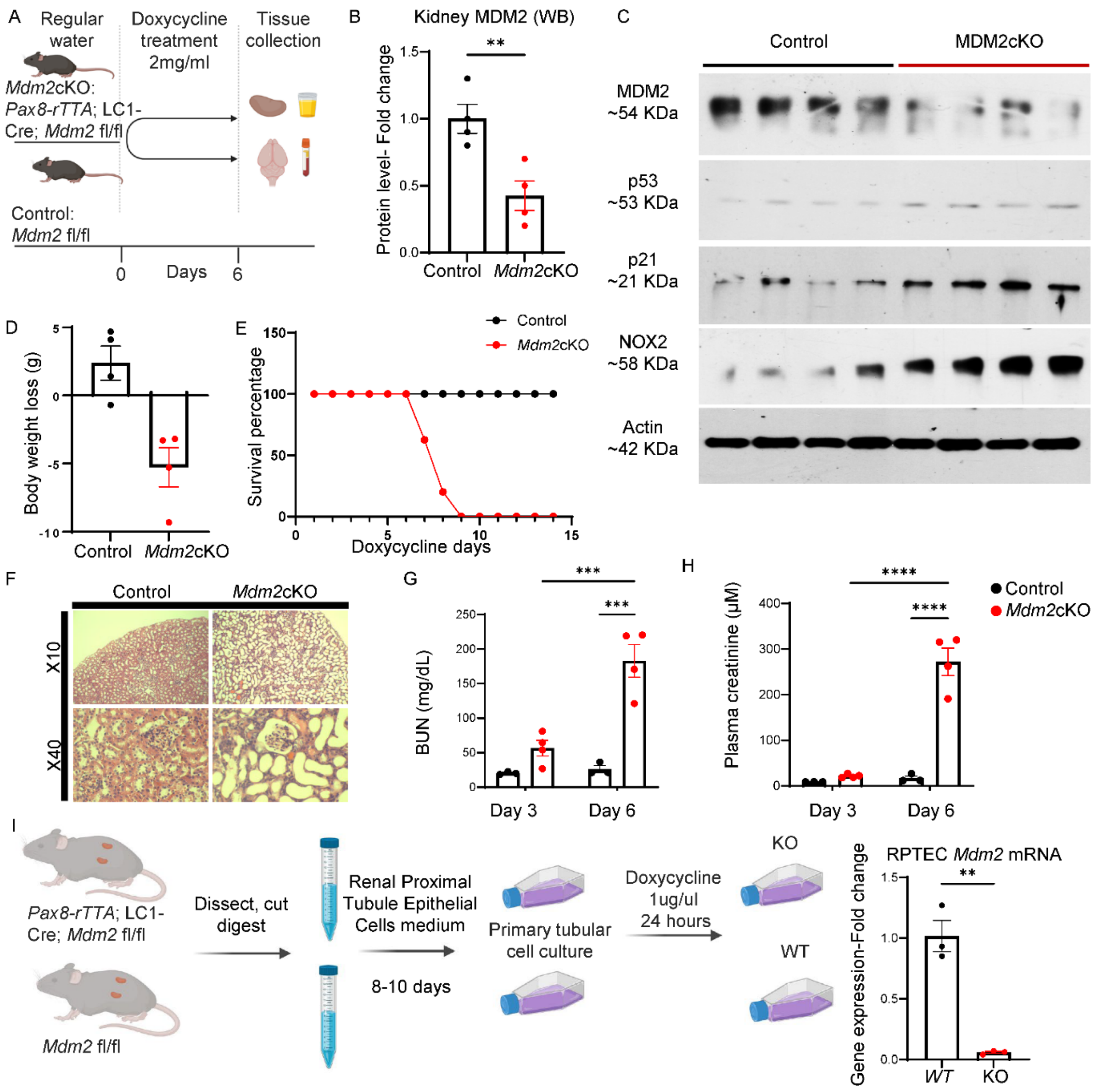

Supplemental Figure 1: Characterization of the *Mdm2* conditional knock-out rapid kidney failure mouse model.

(A) Schematic representation of experimental design in *Pax8*-rtTAcre;*Mdm2* f/f and control *Mdm2* fl/fl mice, both administered doxycycline (2 mg/ml in 5% sucrose drinking water) for tissue and specimen collection on day 6. *Created in BioRender. Saliba, A. (2025) https://BioRender.com/r27y246.* **(B)** Kidney MDM2 protein level. **(C)** Immunoblot showing expression of MDM2 (~54 kDa), p53 (~53 kDa), p21 (~21 kDa), NOX2 (~58 kDa), and Actin (~42 kDa). **(D)** Body weight loss at day 6 of doxycycline treatment (n=4 per group). **(E)** Survival percentage across 15 days of doxycycline n=10 per group **(F)** H&E staining of kidney FFPE sections, comparing control and *Mdm2*cKO mice kidneys at X10 and X40 magnifications using brightfield microscopy. **(G)** Blood urea Nitrogen (BUN) levels and **(H)** plasma creatinine levels at day 3 and day 6 of doxycycline administration, with n=3 control and n=4 *Mdm2*cKO. **(I)** Schematic illustrating the extraction of renal proximal tubule epithelial cells from Pax8-rtTAcre;*Mdm2*f/f or *Mdm2* fl/fl mice, followed by treatment with doxycycline at 1 µg/ml, and cell harvest at 24 hours. *Created in BioRender. Saliba, A. (2025) https://BioRender.com/d66g841.* mRNA levels fold change of *Mdm2* normalized to *Gapdh*. Graphs display means ± SEM. One-way ANOVA-Tukey or two-tailed t- tests ** p<0.01; *** p < 0.001 and **** p < 0.0001.

Supplemental Figure 2: Multi-organ injury assessment in mouse model of rapid kidney failure.

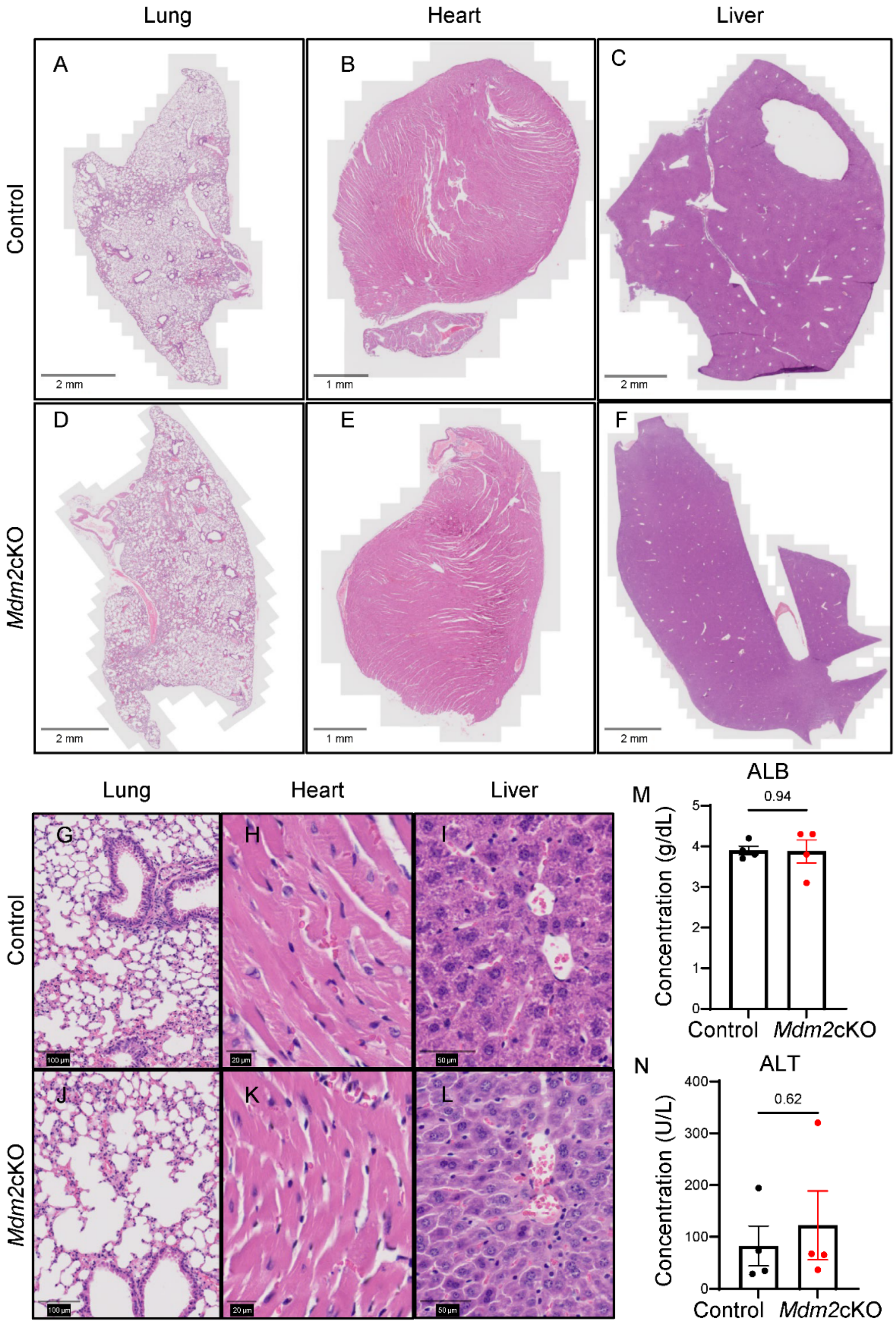

Supplemental Figure 2: Multi-organ injury assessment in mouse model of rapid kidney failure. H&E staining of FFPE sections from **(A)** lung, scale bar= 2 mm, **(B)** heart, scale bar= 1 mm, and **(C)** liver, scale bar=2 mm, harvested at day 6 of doxycycline administration in control *vs*. *Mdm2*cKO mice **(D-F)**. **(G, J)** Zoomed-in images from (A and D respectively); scale bar= 100 µm. **(H-K)** Zoomed-in images from (B and E respectively); scale bar= 20 µm. **(I, L)** Zoomed-in images from (C and F respectively); scale bar= 50 µm. **(M)** Albumin (ALB) and **(N)** alanine aminotransferase (ALT) concentrations in *Mdm2*cKO *vs.* control mice measured with Vetscan-HM5-Hematology Analyzer. Graphs display means ± SEM.

Supplemental Figure 3: Comparative analysis of plasma tryptophan metabolites in *Mdm2cKO vs.* control mice following doxycycline administration at day 3 and day 6.

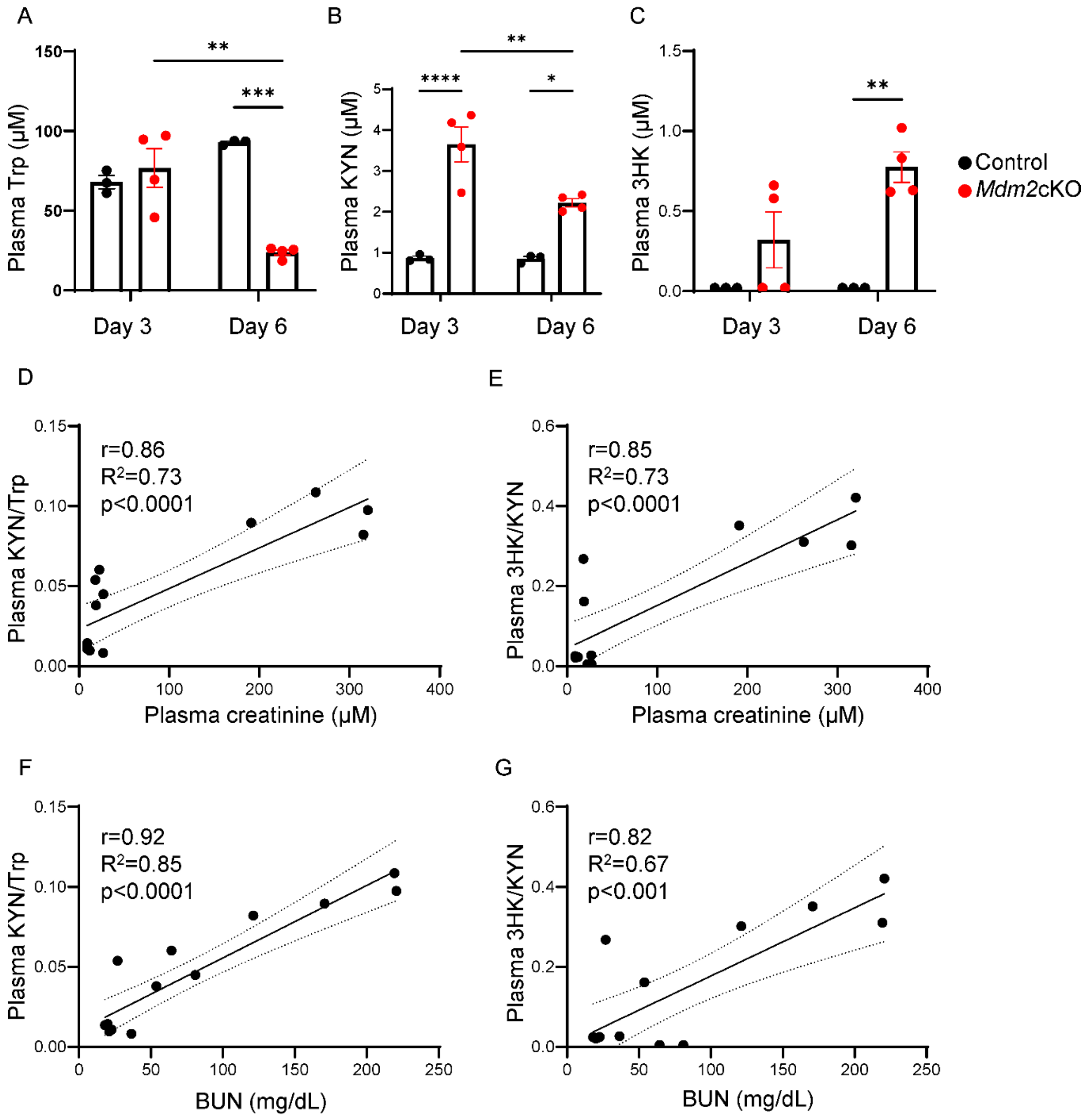

Supplemental Figure 3: Comparative analysis of plasma tryptophan metabolites in *Mdm2cKO vs.* control mice following doxycycline administration at day 3 and day 6.

(**A**) Tryptophan (Trp), **(B)** Kynurenine (KYN), **(C)** 3-hydroxykynurenine (3HK), **(D)** Pearson correlation analysis of KYN/Trp ratio *vs.* plasma creatinine levels **(E)** Pearson correlation analysis of plasma 3HK/KYN ratio *vs.* creatinine levels. **(F)** Pearson correlation analysis of plasma KYN/Trp ratio *vs.* BUN levels (**G)** Pearson correlation analysis of plasma 3HK/KYN ratio *vs.* BUN levels. All correlation analyses include day 3 and day 6 datasets of both control and *Mdm2*cKO mice combined. Graphs display means ± SEM. One-way ANOVA Tukey test; * $p < 0.05$, ** $p < 0.01$, *** $p < 0.001$, and **** $p < 0.0001$.

Supplemental Figure 4: Protein and mRNA analysis of kidney cortex in *Mdm2*cKO *vs.* control mice. (A) mRNA levels of hypoxia-inducible factor 1a (*Hif1a*), **(B)** protein levels of HIF1-α, **(C)** mRNA levels of C-C motif ligand 2 (*Ccl*2), **(D)** protein levels of CCL2, **(E)** mRNA levels of Interleukin 6 (*Il6)*, **(F)** protein levels of IL-6. Graphs display means ± SEM. Two-tailed t- tests ** p<0.01; ***.

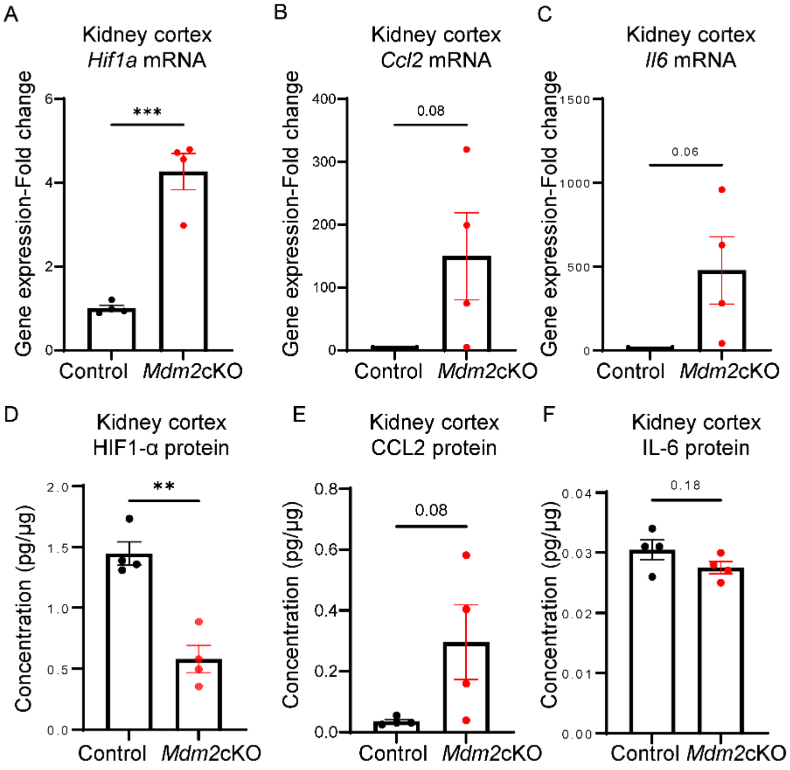

Supplemental Figure 5: Bulk metabolomics of plasma, kidney cortex, and brain cortex in *Mdm2*cKO *vs.* control mice. Data from bulk targeted metabolomics of 32 metabolites in *Mdm2*cKO *vs.* control mice were log-transformed and auto-scaled to account for differences in metabolite concentrations using MetaboAnalyst 5.0. **(A)** plasma, **(B)** kidney cortex and **(C)** brain cortex. PLS-DA PCA plots were generated to show the separation between *Mdm2*cKO mice and control mice based on their metabolic profiles. Each data point represents an individual mouse, with *Mdm2*cKO mice shown in green and control mice shown in red. **(D)** plasma, **(E)** kidney cortex, **(F)** and brain cortex.

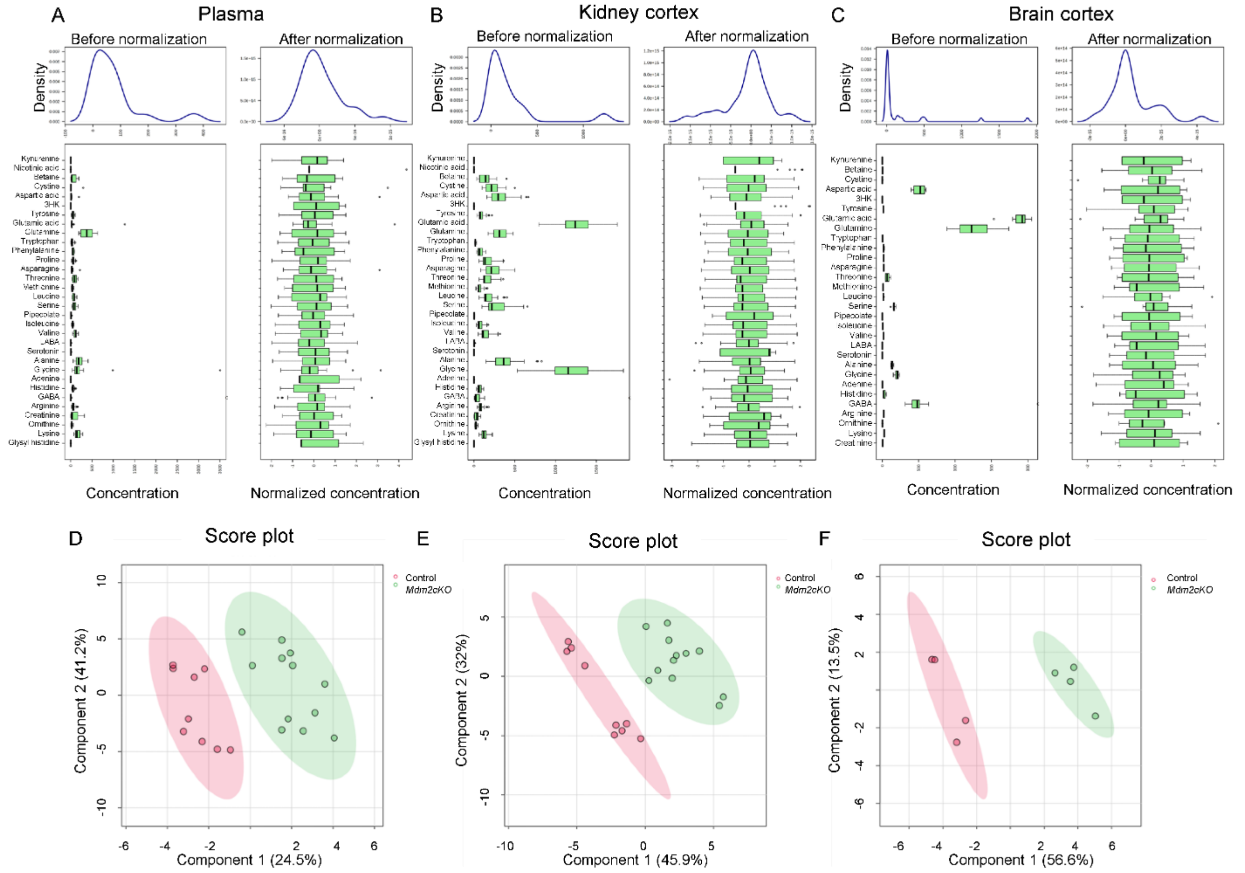

Supplemental Figure 6: Metabolic pathway enrichment analysis reveals altered tryptophan and glutamate metabolism in brain of rapid kidney failure mouse model. Targeted metabolomics and pathway enrichment analysis in the brain cortex of *Mdm2*cKO mice *vs.* control mice involving 32 metabolites. An overview of unbiased pathway enrichment analysis was performed using MetaboAnalyst 5.0 with MBRole2.0 open databases using SMPDB library.

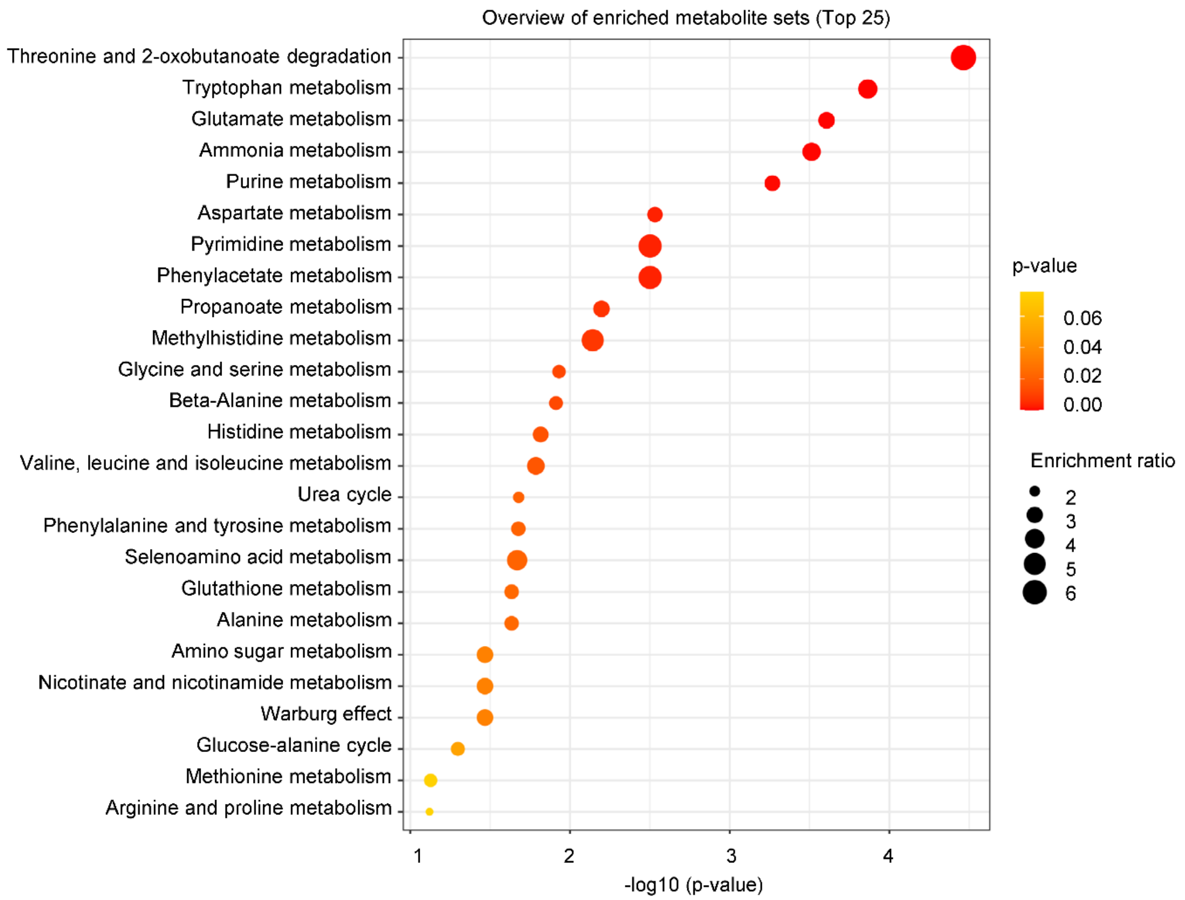

Supplemental Figure 7: Brain MDM2 levels in *Mdm2*cKO *vs.* control mice. (A) Western blot analysis of brain lysate extracted with RIPA buffer (10 µg/well). LICOR imaging of MDM2 protein (54 KDa) in red using IRDye 680RD (emission at 680 nm) and β-actin (42 KDa) in green with IRDye 800CW (emission at 792 nm). The full, unedited blot is presented for n=4 *Mdm2*cKO mice *vs.* n=4 control mice. **(B)** Quantitative analysis of Western blot data, with values normalized to β-actin levels**. (C)** qPCR analysis of brain cortex *Mdm2* mRNA normalized to *Gapdh* mRNA. Graph display means +/- SEM and two-tailed t-test, with * $p < 0.05$; *** $p < 0.001$.

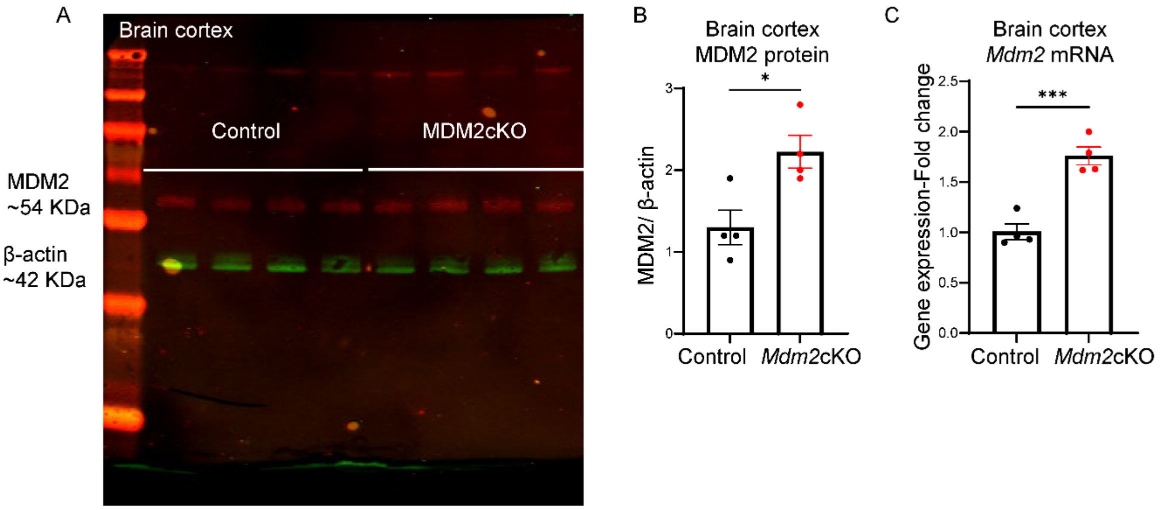

Supplemental Figure 8: Ischemia reperfusion-induced acute kidney injury mouse model. (A) Schematic representation of the ischemia reperfusion (IR) injury experiment. *Created in BioRender. Saliba, A. (2025) https://BioRender.com/x99u069.* **(B)** Plasma creatinine levels, **(C)** blood urea Nitrogen (BUN) levels, **(D)** Pearson correlation between 3-hydroxykynurenine/kynurenine ratio (3HK/KYN) and plasma creatinine and **(E)** Pearson correlation between 3HK/KYN and BUN levels. Graphs display means ± SEM. Two-tailed t-tests **** p < 0.0001.

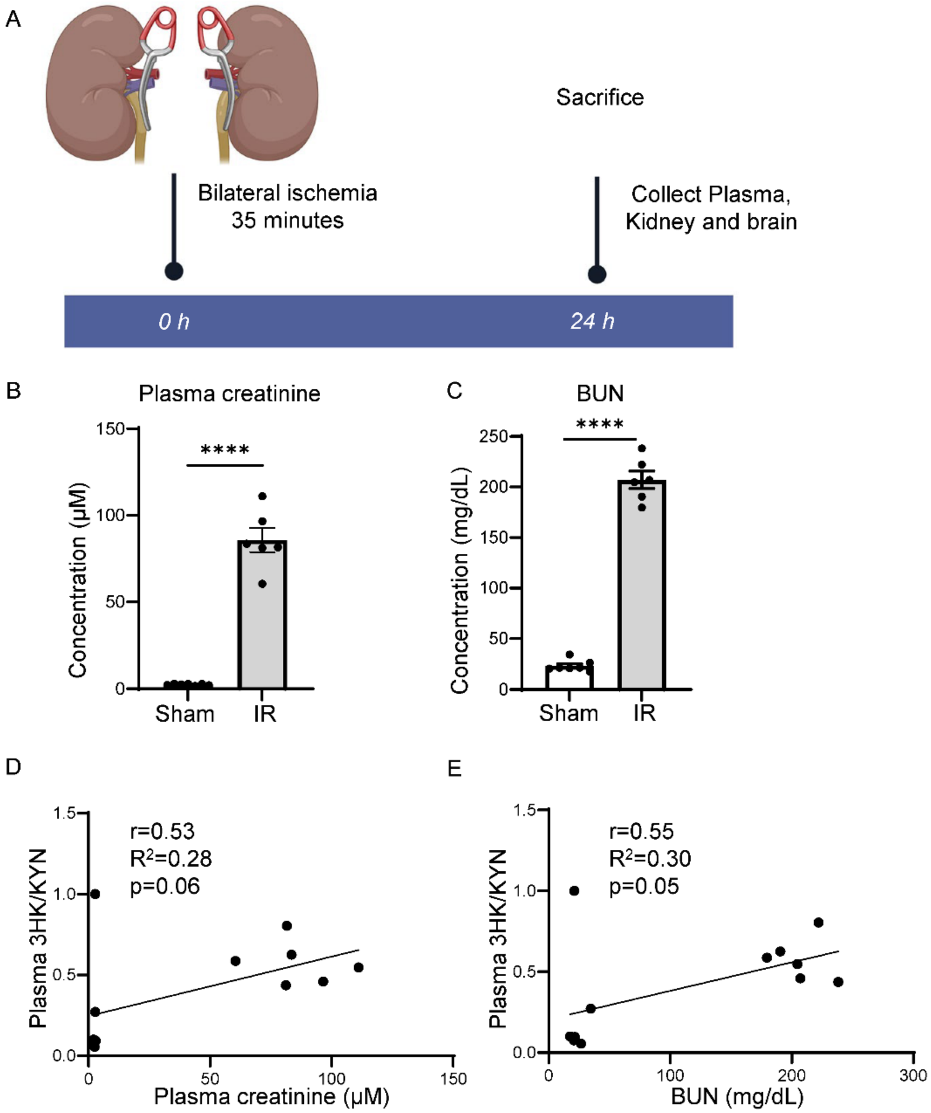

Supplemental Figure 9: Kidney function in adenine-induced chronic kidney disease mouse model. (A) Schematic of the adenine-induced chronic kidney disease (CKD) mouse model. Mice were fed either 0.2% adenine-supplemented food or regular chow for 30 days (n=4 per group). The scheme was *created in BioRender. Saliba, A. (2025) https://BioRender.com/n83d154.* Concentrations of **(B)** urinary albumin-to-creatinine ratio (ACR), **(C)** plasma creatinine, and **(D)** blood urea Nitrogen (BUN). Graphs display means ± SEM. Two-tailed t- tests * p < 0.05; ** p<0.01. Scheme created with Biorender.com.

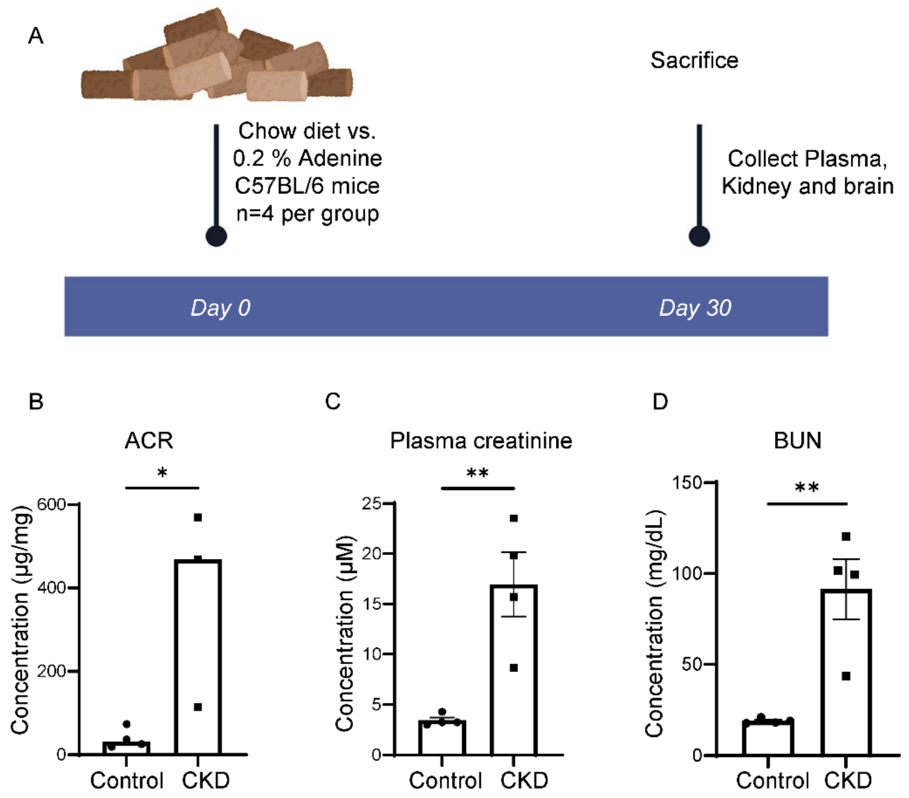

Supplemental Figure 10: Pearson's correlation between markers of kidney function, inflammation and plasma tryptophan metabolites in adenine-induced CKD mouse model.

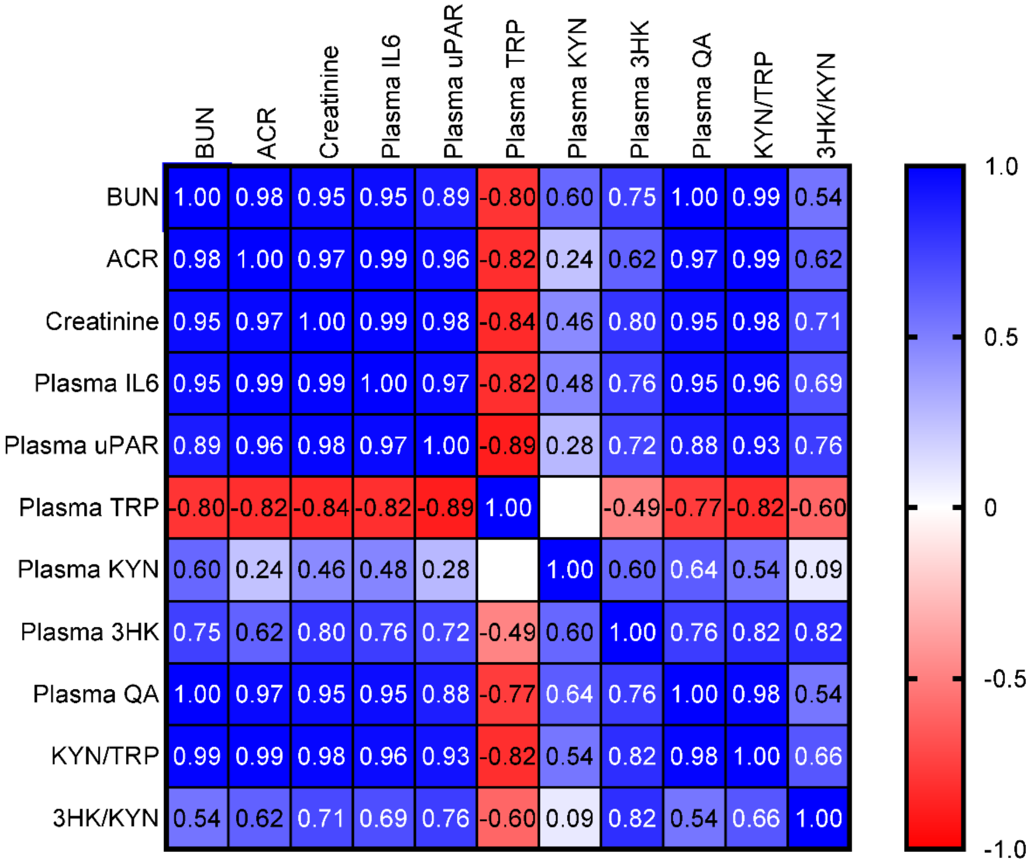

Supplemental Figure 11: Pearson's correlation between markers of kidney function, inflammation and kidney tryptophan metabolites in adenine-induced CKD mouse model.

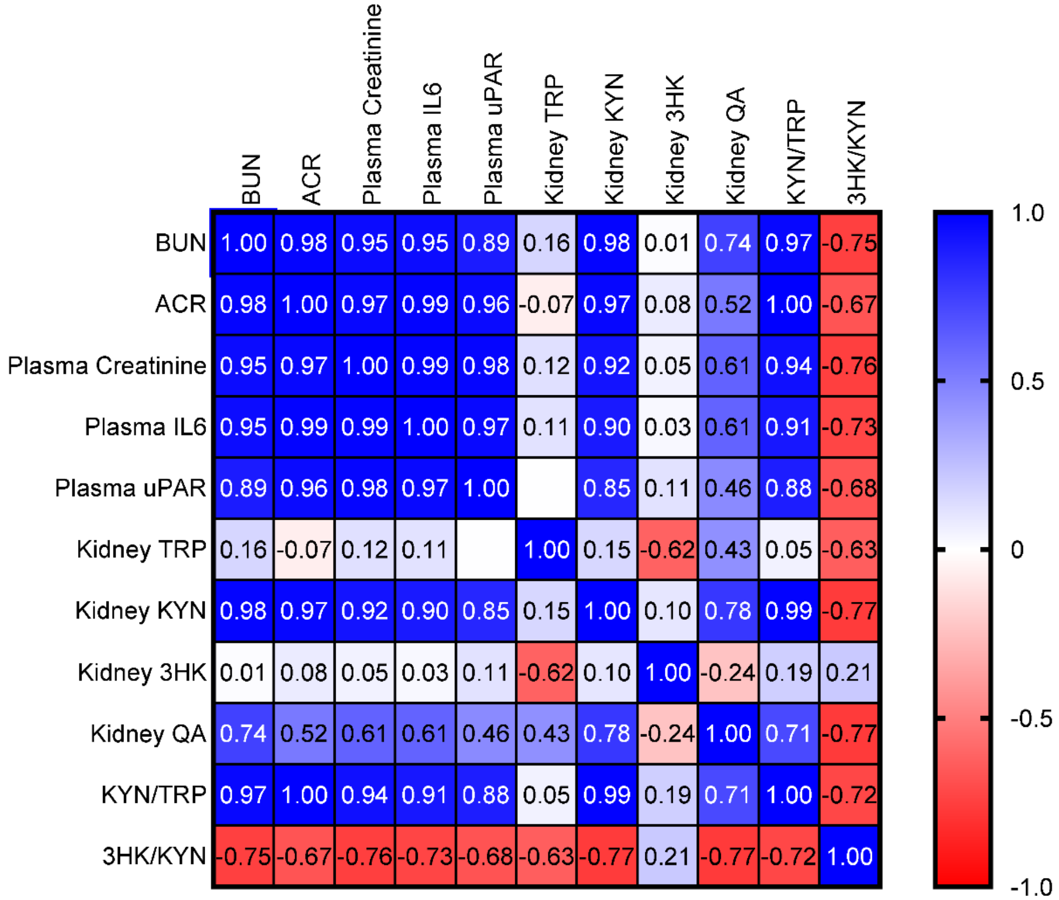

Supplemental Figure 12: Pearson's correlation between markers of kidney function, inflammation and brain tryptophan metabolites in adenine-induced CKD mouse model.

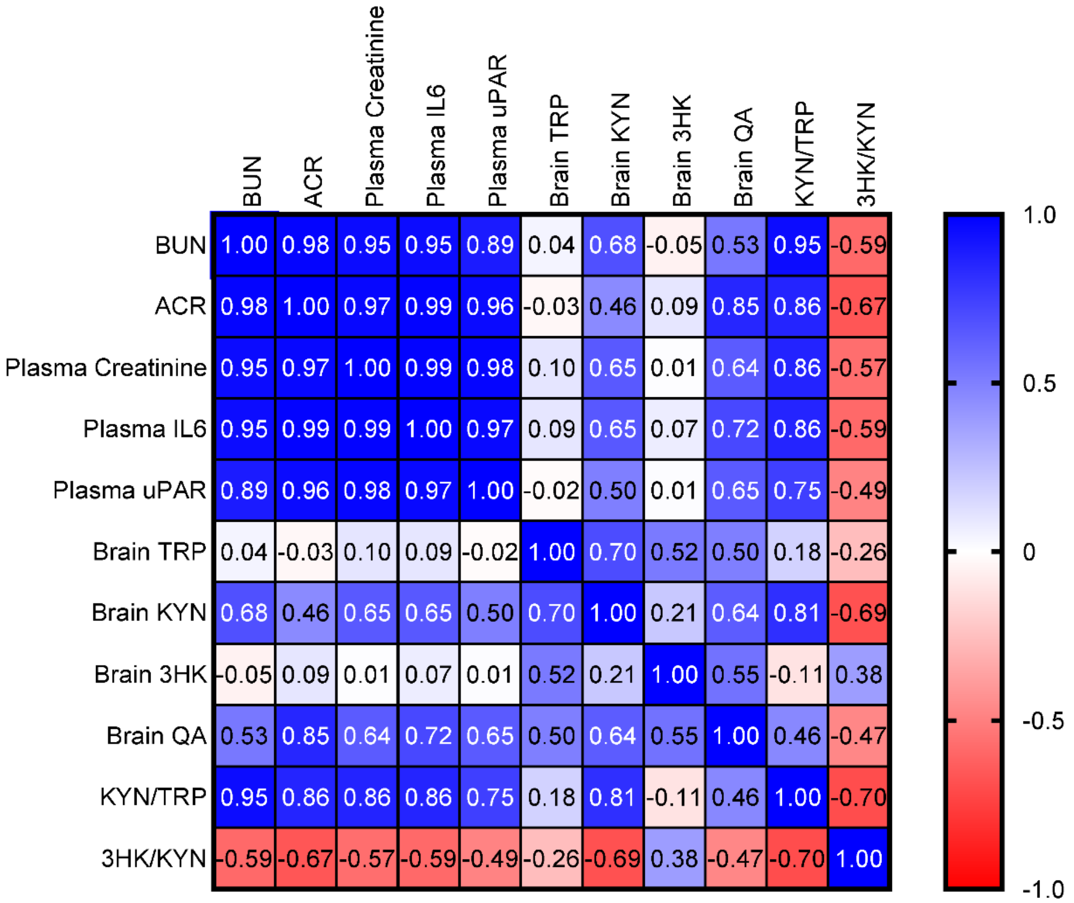

Supplemental Figure 13: Quinolinic acid increases extracellular matrix proteins in human kidney 2 cells. (A) Immunoblots comparing vehicle control and quinolinic acid (QA) treatment at 2.5 μM and 5 μM (n=3 per group) for fibronectin (FN1) (top), collagen, type I, alpha 2 (COL1A2) (middle), and actin (below). **(B-C)** FN1 and COL1A2 band intensity, normalized to actin and presented as fold change to control. Graphs display means ± SEM. One-way ANOVA-Tukey * $p<0.05$, ** $p<0.01$.

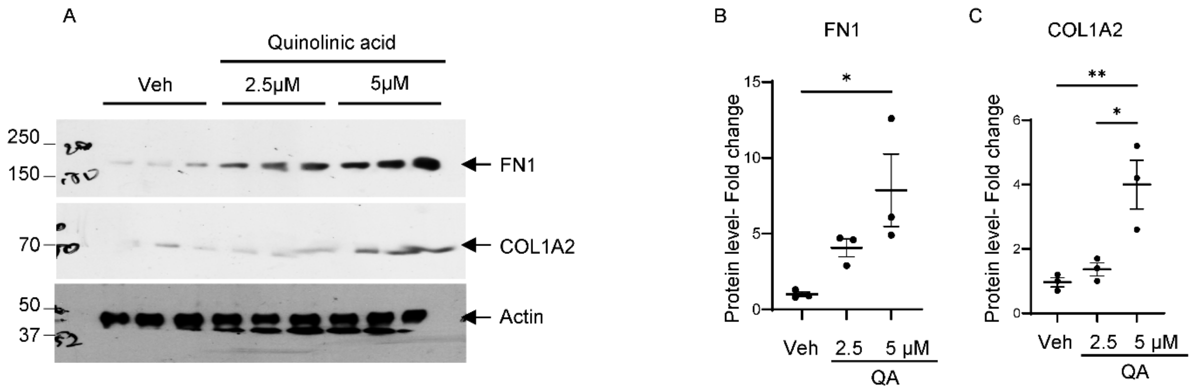

Supplemental Table 1. Pathway enrichment analysis of plasma metabolomics in mice with rapid kidney failure.

Top 5 pathways identified through enrichment analysis using MetaboAnalyst 5.0 and MBRole2.0, incorporating SMPDB, based on targeted bulk metabolomics of 32 plasma metabolites in *Mdm2*cKO *vs.* control mice. (Corresponding to Figure 1 A).

**Pathway**	**Total Cmpd**	**Hits**	**FDR**
Tryptophan Metabolism	60	5	7.52E-03
Selenoamino Acid Metabolism	28	1	2.52E-01
Methylhistidine Metabolism	4	1	2.52E-01
Catecholamine Biosynthesis	20	1	2.52E-01
Thyroid hormone synthesis	13	1	2.52E-01

Supplemental Table 2. Pathway enrichment analysis of brain metabolomics in mice with rapid kidney failure.

Top 5 pathways identified through enrichment analysis using MetaboAnalyst 5.0 and MBRole2.0, incorporating SMPDB, based on targeted bulk metabolomics of 32 metabolites in brain cortex of *Mdm2*cKO *vs.* control mice. (Corresponding to Supplemental Figure 6).

**Pathway**	**Total Cmpd**	**Hits**	**FDR**
Threonine and 2-Oxobutanoate Degradation	20	1	1.34E-03
Tryptophan Metabolism	60	5	2.66E-03
Glutamate Metabolism	49	6	2.99E-03
Ammonia Recycling	32	6	2.99E-03
Purine Metabolism	74	5	4.21E-03

Supplemental Table 3. Characteristics of the study participants.

Variables (mean ± SD or N (%))	All (n=26)	CKD 3b-4 (n=18)	CKD 5 (n=8)
**Age, yr**	57.08 ± 10.21	57.06 ± 9.08	57.13 ± 13.12
**Female, n**	8 (30.77%)	5 (27.78%)	3 (37.5%)
Body mass index, (kg/m^2)	34.66 ± 7.72	35.32 ± 9.13	33.18 ± 2.58
Systolic blood pressure, mmHg	147.35 ± 19.35	144.22 ± 19.59	154.38 ± 17.98
Diastolic blood pressure, mmHg	75.73 ± 13.04	76.50 ± 12.28	74.00 ± 15.36
**Serum creatinine, mg/dL**	3.01 ± 1.70	2.03 ± 0.6	5.22 ± 1.19 $
**eGFR, mL/min/1.73m^2**	28.04 ± 13.60	35.83 ± 7.73	10.5 ± 2.67 #
Blood hemoglobin, g/dL	11.25 ± 2.15	11.79 ± 1.84	10.05 ± 2.41
Glycated hemoglobin, %	7.1 ± 1.1	7.5 ± 0.8	6.2 ± 1.4 *
Urine albumin creatinine ratio, mg/g	3715.65 ± 3038.78	2462.39 ± 2604.92	6535.50 ± 1836.72
eGFR, estimated glomerular filtration rate			

Two-tailed t-test: *$p<0.05$ $^{\$}p < 1x10^{-6}$; $^{\#}p<1x10^{-8}$

Supplemental Table 4. **Plasma-free levels of tryptophan and selective tryptophan metabolites.**

Metabolites (mean ± SD)	All (n=26)	CKD 3b-4 (n=18)	CKD 5 (n=8)
**Tryptophan, µm/L**	71.56 ± 22.34	81.04 ± 19.98	50.22 ± 7.86 ***
**Kynurenine, µm/L**	7.21 ± 2.01	7.28 ± 2.08	7.06 ± 1.95
**Kynurenic acid, µm/L**	0.36 ± 0.20	0.29 ± 0.16	0.52 ± 0.21 **
**3-Hydroxykynurenine, µm/L**	0.03 ± 0.04	0.01 ± 0.03	0.04 ± 0.06
**3-Hydroxyanthranilic acid, µm/L**	6.20 ± 8.64	6.30 ± 10.17	5.98 ± 3.93
**Quinolinic acid, µm/L**	1.97 ± 2.42	1.13 ± 0.95	3.87 ± 3.56 **
**Kynurenine/Tryptophan**	0.11 ± 0.04	0.09 ± 0.03	0.14 ± 0.02 **
**3Hydroxykynurenine/Kynurenine**	0.003 ± 0.004	0.001 ± 0.002	0.005 ± 0.005 *
**Quinolinic acid/Tryptophan**	0.03 ± 0.04	0.015 ± 0.013	0.074 ± 0.057 ***

Two-tailed t-test: * $p < 0.05$; ** $p < 0.01$; *** $p < 0.001$

Supplemental Table 5. Self-reported scores of brief fatigue inventory (BFI).

Questionnaire (mean ± SD)	All (n=26)	CKD 3b-4 (n=18)	CKD 5 (n=8)
**BFI (total score)**	5.15 ± 3.08	4.79 ± 2.99	5.69 ± 3.36

Supplemental Table 6. Primers list for mouse genotyping.

Primers	Sequence
FM-A	5’-TGTGGAGAAACAGTTACTTC
FM-B	5’- CTGTGCTCCTTCACAGAG
FM-C	5’TGAGATGAGTCAAAGCCTGG
Pax8-rtTA-F	CCATGTCTAGACTGGACAAGA
Pax8-rtTA-R	CTCCAGGCCACATATGATTAG
LC1/Cre-F	TCGCTGCATTACCGGTCGATGC
LC1/Cre-R	CCATGAGTGAACGAACCTGGTCG

Supplemental Table 7. qPCR primers list.

Gene	Forward sequence	Reverse sequence	Reference or Catalog number
Mouse_*Gapdh* (IDT*)	TGTGTCCGTCGTGGATCTGA	CCTGCTTCACCACCTTCTTGAT	(1)
Mouse_*Cxcl1* (IDT)	TCTCCGTTACTTGGGGAC	CCACACTCAAGAATGGTCGC	(2)
Mouse_*Tnf* (IDT)	CGTCAGCCGATTTGCTATCT	CGGACTCCGCAAAGTCTAAG	(3)
Mouse_*Nox2* (IDT)	CCCTTTGGTACAGCCAGTGAAGAT	CAATCCCGGCTCCCACTAACATCA	(4)
Mouse_*Mdm2* (IDT)	GGAGTCCCGAGTTTCTCTGTG	CTTGCTGACTTACAGCCACTAAA	Detect exons 5,6 of the *Mdm2* gene
Mouse_*Hif1a*	GAA CAT CAA GTC AGC AAC GTG	TTT GAC GGA TGA GGA ATG GG	PMID: 25909079
Mouse_*Cdkn1a*	NM_007669 RT² qPCR Primer Assay for Mouse Cdkn1a, GeneGlobe ID: PPM02901B-200		Qiagen Cat#330001
Mouse_*Ccl2*	NM_011333 RT² qPCR Primer Assay for Mouse Ccl2, GeneGlobe ID - PPM03151G-200		Qiagen Cat# 330001
Mouse_*Il1b*	NM_008361 RT² qPCR Primer Assay for Mouse Il1b, GeneGlobe ID - PPM03109F-200		Qiagen Cat#330001
Mouse_*Il6*	NM_001314054 RT² qPCR Primer Assay for Mouse Il6, GeneGlobe ID - PPM03015A-200		Qiagen Cat#330001
Mouse_*Trp53*	NM_022654 RT2 PCR Primer Set for Mouse Trp53tg5, Gene ID: PPM32034A-200		Qiagen Cat#330001

*IDT: Integrated DNA Technologies; Glyceraldehyde-3-phosphate dehydrogenase (*Gapdh)*

Supplemental Table 8. Stages of chronic kidney disease based on eGFR categories.

Stage	Description	eGFR (mL/min/1.73m^2)
G1	Normal or high	≥90
G2	Mildly decreased	60-89
G3a	Mild to moderately decreased	45-59
G3b	Moderately to severely decreased	30-44
G4	Severely decreased	15-29
G5	Kidney failure	<15

Reference: (5).

Supplemental Methods

Primary renal tubular epithelial cells (pTECs): The isolation of pTECs was performed using *Pax8*-rtTAcre;*Mdm2*f/f or *Mdm2*f/f control mice (8-10 weeks old) following established protocols (6) with slight modifications as previously described (7). After cervical dislocation, both kidneys were extracted, and the cortical regions were finely chopped and washed with PBS containing 10% Penicillin-Streptomycin (Thermo Fisher Scientific). Collagenase IV (Sigma) treatment was applied for 30 minutes at 37°C with gentle shaking. The digested tissue was then filtered through a 70 µm filter and resuspended in renal epithelial growth medium (REGM). The suspension was centrifuged at 50 g for 5 minutes at room temperature (RT), and the resulting pellet was resuspended in 5 ml of REGM. After centrifugation of the supernatant at 50 g for 5 minutes at RT, the second pellet containing tubular cells was resuspended in 5 ml of REGM. Both pellet suspensions were combined (total of 20 ml) and centrifuged again at 50 g for 5 minutes at RT. The final pellet was resuspended in 2 ml of REGM and plated onto a collagen coated T25 flask. Cells were cultured under 5% CO2 at 37°C, with media changed every 48 hours. After 8-10 days, tubular cells were passaged for further experiments, and all studies were performed using passage 1 cells. pTECs isolated from *Pax8*-rtTAcre;*Mdm2*f/f or *Mdm2*f/f control mice were treated for 24 h with 1µg/ml doxycycline to induce *Mdm2* depletion (8).

Protein extraction and western blot from brain tissues: Protein extraction was done using RIPA buffer followed by quantification using BCA assay (Thermofisher, #23235), and equal protein quantities were loaded (12 µl). Transfer on PVDF membranes was followed by blocking (LICOR #927-60001) and incubation with 1:500 primary mouse-anti-MDM2 (Fishe scientific; #PIMA124643) or 1:5000 β-Actin Antibody (Cell signaling, #4967) and 1:10,000 secondary antibodies i.e., goat anti-Rabbit IgG (LICOR, #926-32211) and goat anti-mouse IgG (LICOR, #926-68070). Protein bands were visualized using LICOR Odyssey CLx.

Quinolinic acid effect on Human proximal tubular epithelial cells (HK2): HK2 cells were purchased from ATCC (#CRL-2190) and cultured in DMEM/F12 medium supplemented with 10% fetal bovine serum. Cells were plated and allowed to reach confluence before being serum-starved in serum-free medium for 24 hours. After starvation, the cells were treated with quinolinic acid (Cayman chemicals #14941) at concentrations of 2.5 μM and 5 μM for 24 hours. Following treatment, cells were lysed using RIPA buffer (Abcam, #ab156034) with protease and phosphatase inhibitor (Fisher Scientific, #78440) and quantified by BCA assay (Fisher Scientific, #23235). For immunoblotting, protein lysates were separated by SDS-PAGE, transferred to PVDF membranes, and incubated with primary antibodies (1:1000) overnight at 4°C, i.e., Actin (C-2) (Santa Cruz Biotechnology, #sc-8432); COL1A2 (G-4) (Santa Cruz Biotechnology, #sc-166865); Fibronectin (Sigma Aldrich, #F3648). Membranes were probed with horseradish peroxidase-conjugated secondary antibody (1:10,000), followed by ECL detection. Band intensities were quantified using ImageJ corrected for background and normalized to Actin intensity.

Enzyme-linked immunosorbent assay (ELISA) protein quantifications: Protein extraction from kidney cortex was performed using RIPA buffer (Abcam, #ab156034) with phosphatase and protease inhibitor cocktail (Fisher scientific, #78440). Total protein concentration was quantified using the BCA protein assay kit (Thermo Fisher Scientific, #23235). Quantitative protein levels in urine and tissue lysates were assessed using ELISA kits following manufacturer's protocols. Mouse CCL2/JE/MCP-1 Quantikine ELISA Kit (R&D, #MJE00B), Human/Mouse Total HIF-1 alpha/HIF1A DuoSet IC ELISA (R&D, #DYC1935-2), Mouse IL-6 Quantikine ELISA Kit (R&D, #M6000B-1). For urine Albumin-to-Creatinine-Ratio (ACR): Mouse Albumin ELISA Kit (Bethyl Laboratories, #E99-134), and Creatinine Colorimetric Detection Kit (ENZO; #ADI-907-030A).

Supplemental References

1. Han WK, Kim EH, Shin SA, Shin DS, Kim BJ, Lyu AR, et al. Susceptibility of Diabetic Mice to Noise Trauma. *Biomed Res Int.* 2018;2018:7601232.
2. Schwartz AJ, Converso-Baran K, Michele DE, and Shah YM. A genetic mouse model of severe iron deficiency anemia reveals tissue-specific transcriptional stress responses and cardiac remodeling. *J Biol Chem.* 2019;294(41):14991-5002.
3. Orhue V, Kanaji A, Caicedo MS, Virdi AS, Sumner DR, Hallab NJ, et al. Calcineurin/nuclear factor of activated T cells (NFAT) signaling in cobalt-chromium-molybdenum (CoCrMo) particles-induced tumor necrosis factor-alpha (TNFalpha) secretion in MLO-Y4 osteocytes. *J Orthop Res.* 2011;29(12):1867-73.
4. Santana-Garrido A, Reyes-Goya C, Fernandez-Bobadilla C, Blanca AJ, Andre H, Mate A, et al. NADPH oxidase-induced oxidative stress in the eyes of hypertensive rats. *Mol Vis.* 2021;27:161-78.
5. Stevens PE, Levin A, and Kidney Disease: Improving Global Outcomes Chronic Kidney Disease Guideline Development Work Group M. Evaluation and management of chronic kidney disease: synopsis of the kidney disease: improving global outcomes 2012 clinical practice guideline. *Ann Intern Med.* 2013;158(11):825-30.
6. Ding W, Yousefi K, and Shehadeh LA. Isolation, Characterization, And High Throughput Extracellular Flux Analysis of Mouse Primary Renal Tubular Epithelial Cells. *J Vis Exp.* 2018(136).
7. Darshi M, Tumova J, Saliba A, Kim J, Baek J, Pennathur S, et al. Crabtree effect in kidney proximal tubule cells via late-stage glycolytic intermediates. *iScience.* 2023;26(4):106462.

8. Thomasova D, Ebrahim M, Fleckinger K, Li M, Molnar J, Popper B, et al. MDM2 prevents spontaneous tubular epithelial cell death and acute kidney injury. *Cell Death Dis.* 2016;7(11):e2482.
