# Supplementary material for: Quinolinic acid potentially links kidney injury to brain toxicity
Source: JCI Insight. 2025 Feb 13;10(6):e180229. doi: 10.1172/jci.insight.180229 (PMC11949017; doi:10.1172/jci.insight.180229)
Supplement: Unedited blot and gel images [file jciinsight-10-180229-s070.pdf]

Full unedited gel for Supplemental Figure 1 C.

- **5 blots**

First 4 lanes: n=4 Control mice kidney cortex lysates

Last 4 lanes: n=4 MDM2cKo mice kidney cortex lysates

Full unedited gel for Supplemental Figure 1 C. Blot 1/5

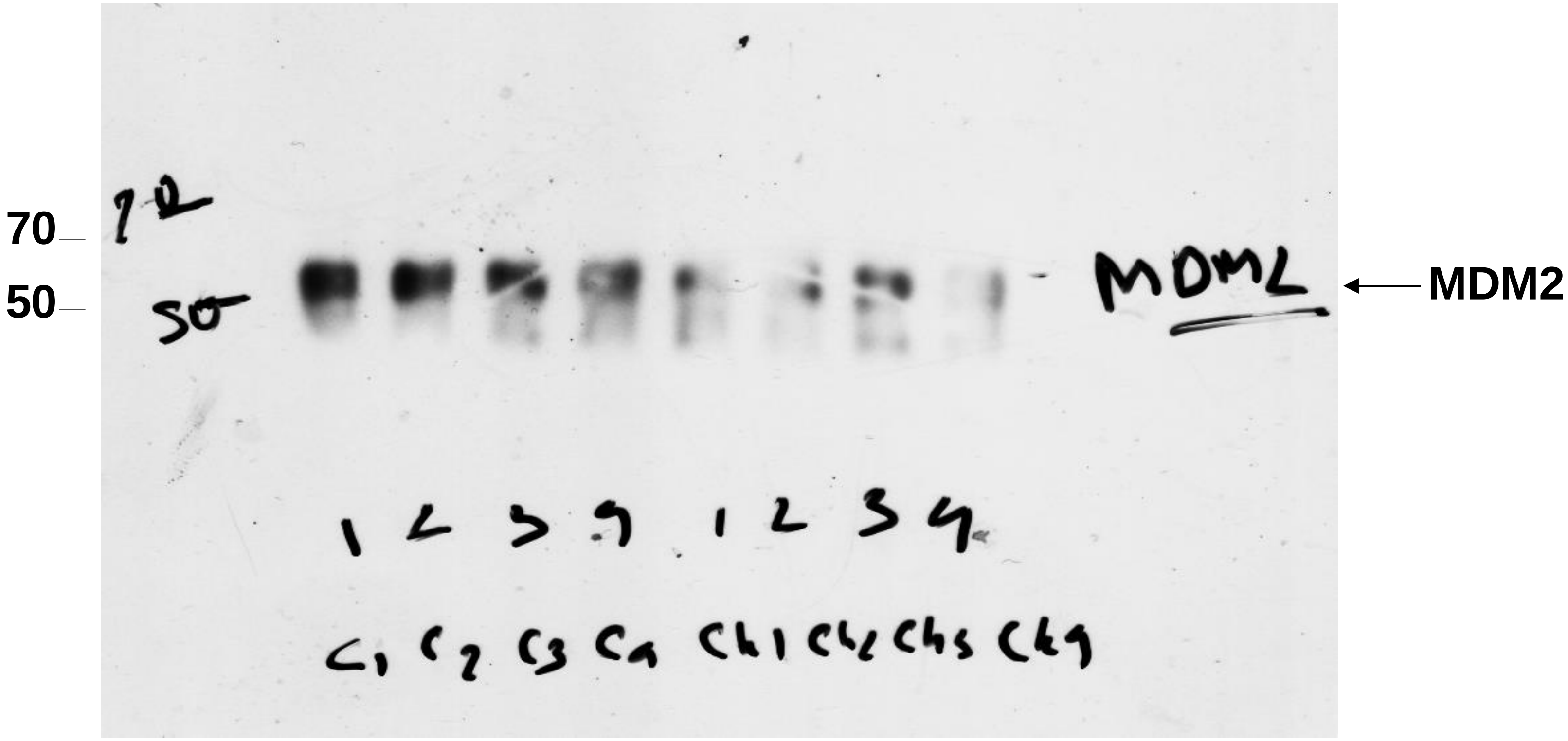

Primary antibodies: (Mouse-anti-MDM2 (ThermoFisher, #MA1-24643); 1:1000

Secondary antibodies: Horseradish peroxidase–conjugated secondary antibody (1:10,000)

ECL reagent was used for detecting the protein signal by exposing it to X-ray film in a dark room to visualize the specific protein band recognized by the primary antibody

Full unedited gel for Supplemental Figure 1 C. Blot 2/5

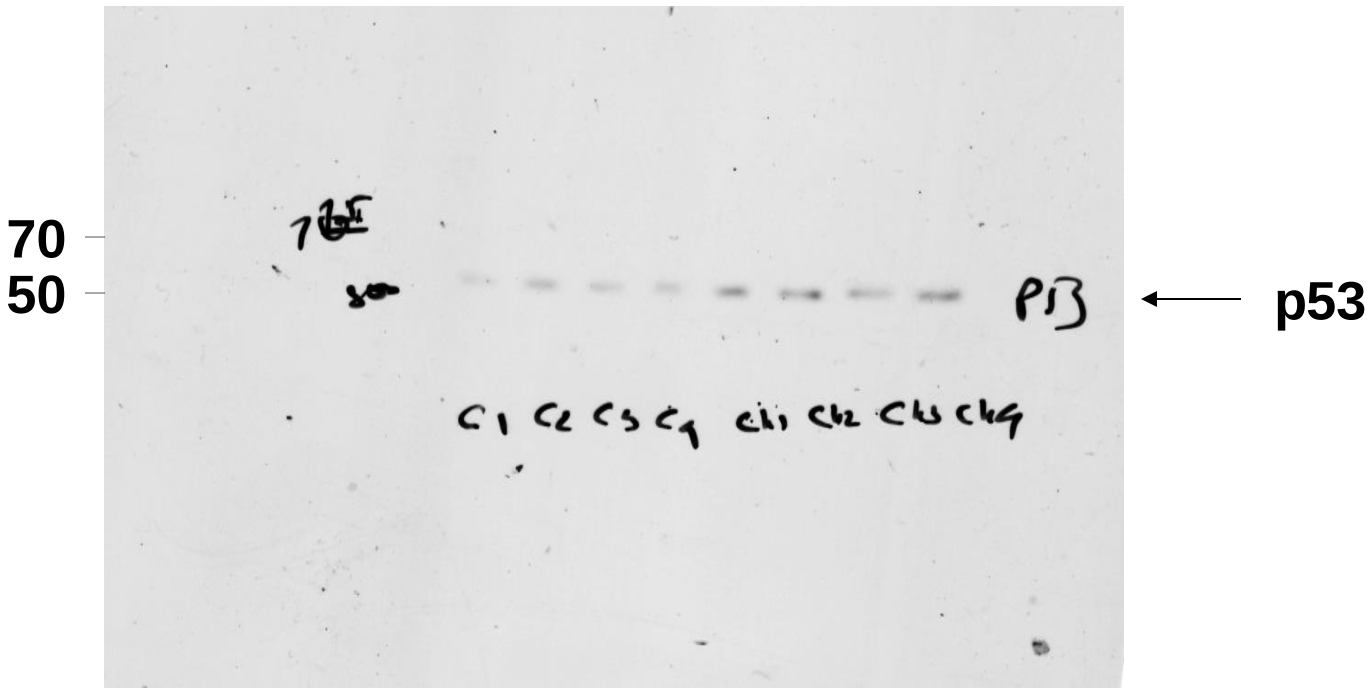

Primary antibodies: Mouse monoclonal IgG2a κ p53 antibody; Santa Cruz Biotechnology, #sc-126; 1:1000

Secondary antibodies: Horseradish peroxidase–conjugated secondary antibody (1:10,000)

ECL reagent was used for detecting the protein signal by exposing it to X-ray film in a dark room to visualize the specific protein band recognized by the primary antibody

Full unedited gel for Supplemental Figure 1 C. Blot 3/5

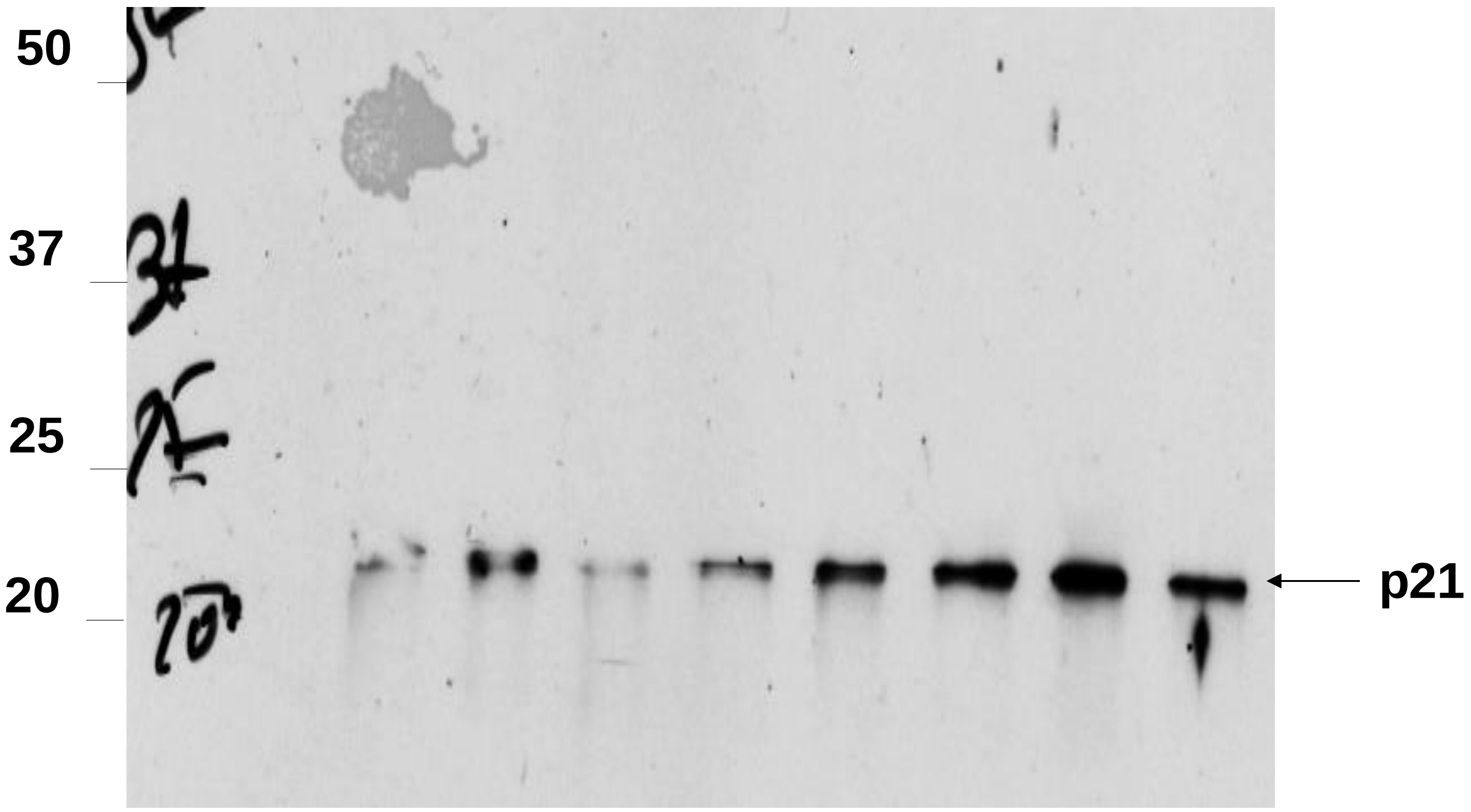

Primary antibodies: Rabbit Recombinant Monoclonal p21 antibody; Abcam, #ab109199; 1:1000

Secondary antibodies: Horseradish peroxidase–conjugated secondary antibody (1:10,000)

ECL reagent was used for detecting the protein signal by exposing it to X-ray film in a dark room to visualize the specific protein band recognized by the primary antibody

Full unedited gel for Supplemental Figure 1 C. Blot 4/5

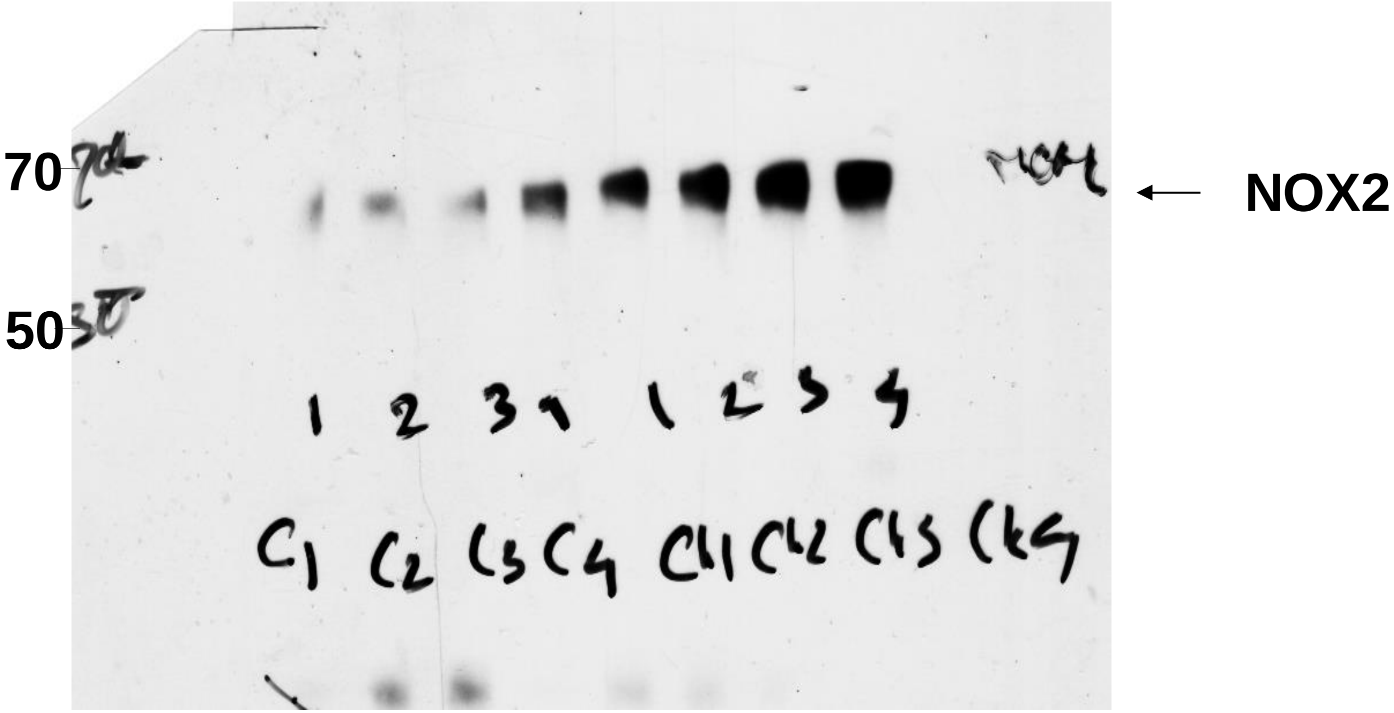

Primary antibodies: NOX2 Recombinant Rabbit Monoclonal Antibody; ThermoFisher, #MA5-35348; 1:1000

Secondary antibodies: Horseradish peroxidase–conjugated secondary antibody (1:10,000)

ECL reagent was used for detecting the protein signal by exposing it to X-ray film in a dark room to visualize the specific protein band recognized by the primary antibody

Full unedited gel for Supplemental Figure 1 C. Blot 5/5

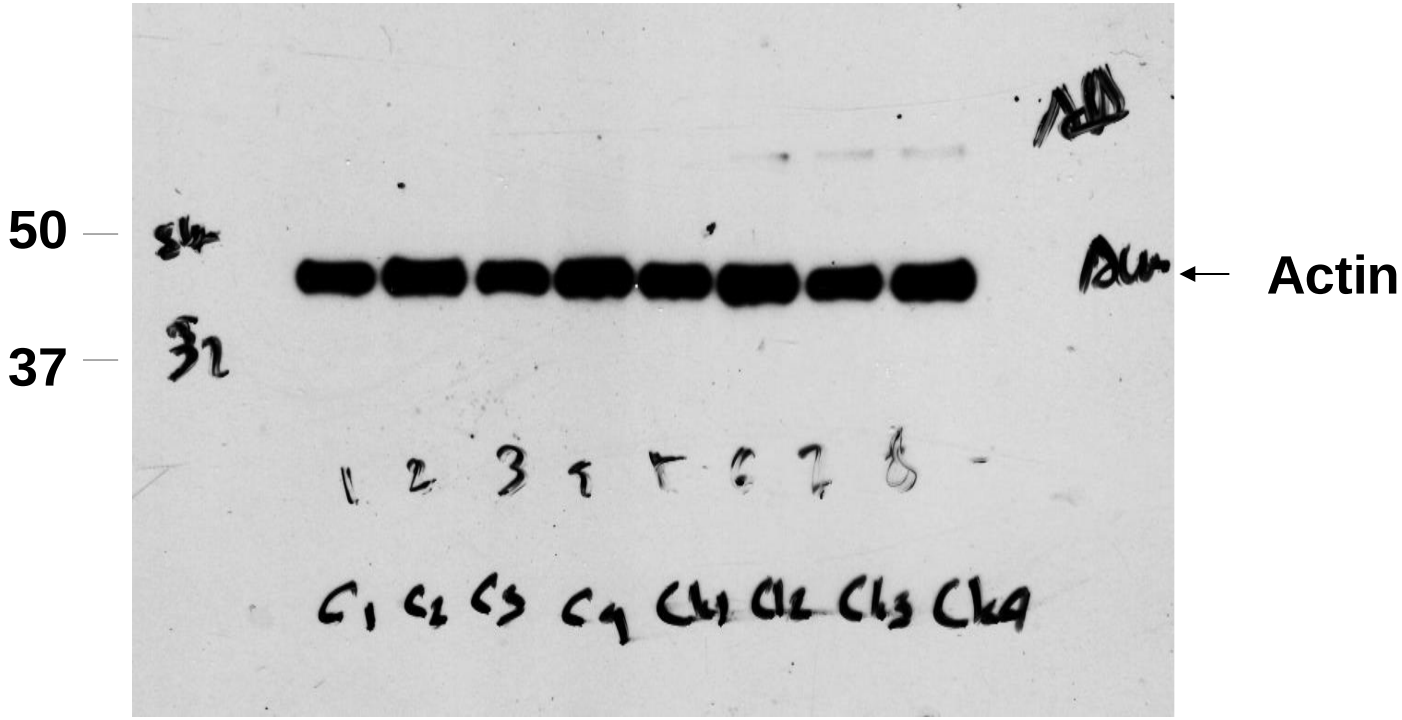

Primary antibodies: Mouse monoclonal IgG1 κ Actin antibody; Santa Cruz Biotechnology, #sc-8432; 1:1000

Secondary antibodies: Horseradish peroxidase–conjugated secondary antibody (1:10,000)

ECL reagent was used for detecting the protein signal by exposing it to X-ray film in a dark room to visualize the specific protein band recognized by the primary antibody

Full unedited gel for Supplemental Figure 7 A.

- **1 blot**

First 4 lanes: Control mice brain cortex lysates

Last 4 lanes: *Mdm2*cKo mice brain cortex lysates

Full unedited gel for Supplemental Figure 7A

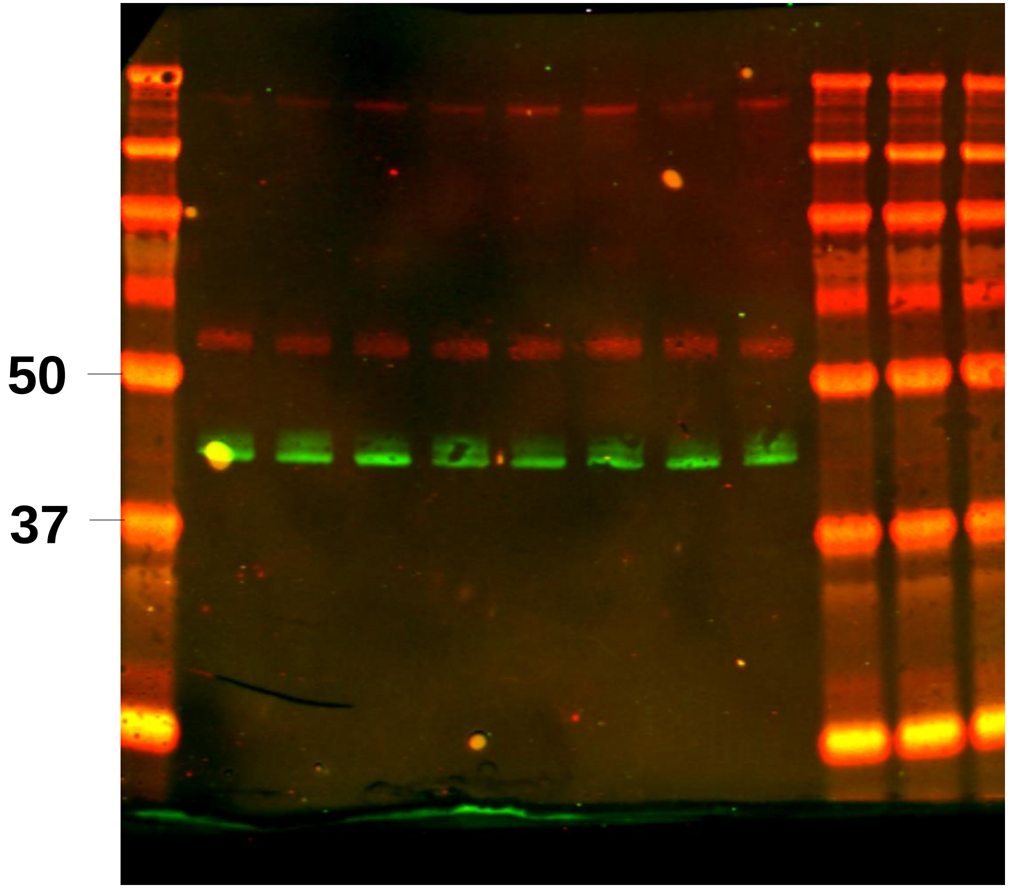

Primary antibodies: Mouse-anti-MDM2 (Thermo Fisher Scientific # MA1-24643) 1:500 or β-Actin Antibody (Cell Signaling # 4967) 1:5000).
Secondary antibodies: Goat anti-rabbit IgG (LICOR #926-32211) and goat anti-mouse IgG (LICOR #926-68070) 1:10,000).

LICOR imaging of MDM2 protein (54 KDa) in red using IRDye 680RD (emission at 680 nm) and β-Actin (42 KDa) in green with IRDye 800CW (emission at 792 nm).

Full unedited gel for Supplemental Figure 13A.

- **3 blots**

Each blot:

First 3 lanes: n=3 HK2 cells + Vehicle

Next 3 lanes: n=3 HK2 cells + 2.5 µM quinolinic acid

Last 3 lanes: n=3 HK2 cells + 5µM quinolinic acid

The human proximal tubular epithelial cells HK2 were purchased from ATCC and grown in DMEM/F12 medium in the presence of 10% fetal bovine serum. The cells were plated and confluent cells were starved in serum-free medium for 24 h prior to incubation with quinolinic acid (Cayman chemicals #14941) at indicated concentration for 24 hours. Cell lysates were immunoblotted with indicated antibodies.

Full unedited gel for Supplemental Figure 13A. Blot 1/3

250

150

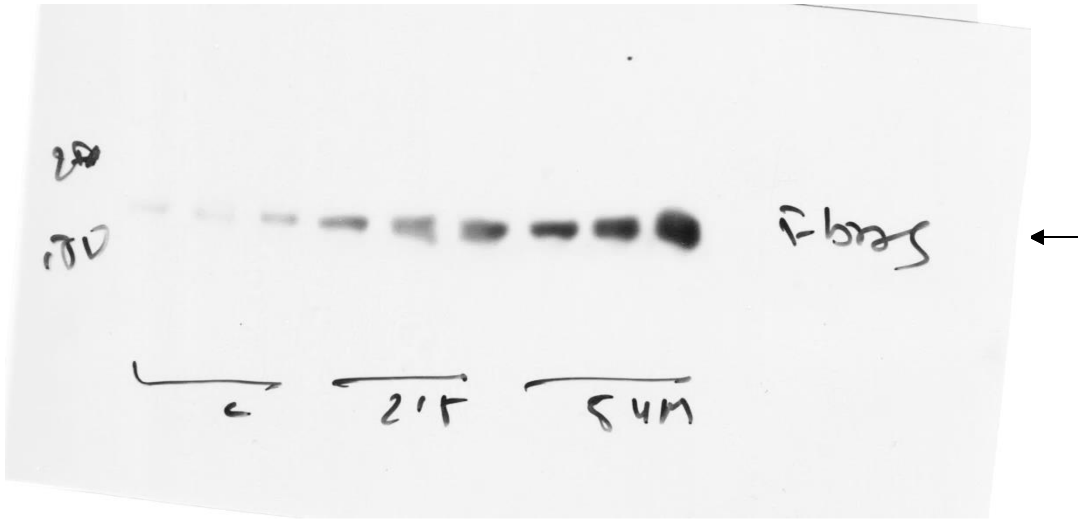

← Fibronectin

Primary antibodies: Rabbit anti-Fibronectin; Sigma-Aldrich, # F3648; 1:1000

Secondary antibodies: Horseradish peroxidase–conjugated secondary antibody (1:10,000)

ECL reagent was used for detecting the protein signal by exposing it to X-ray film in a dark room to visualize the specific protein band recognized by the primary antibody

Full unedited gel for Supplemental Figure 13A. Blot 2/3

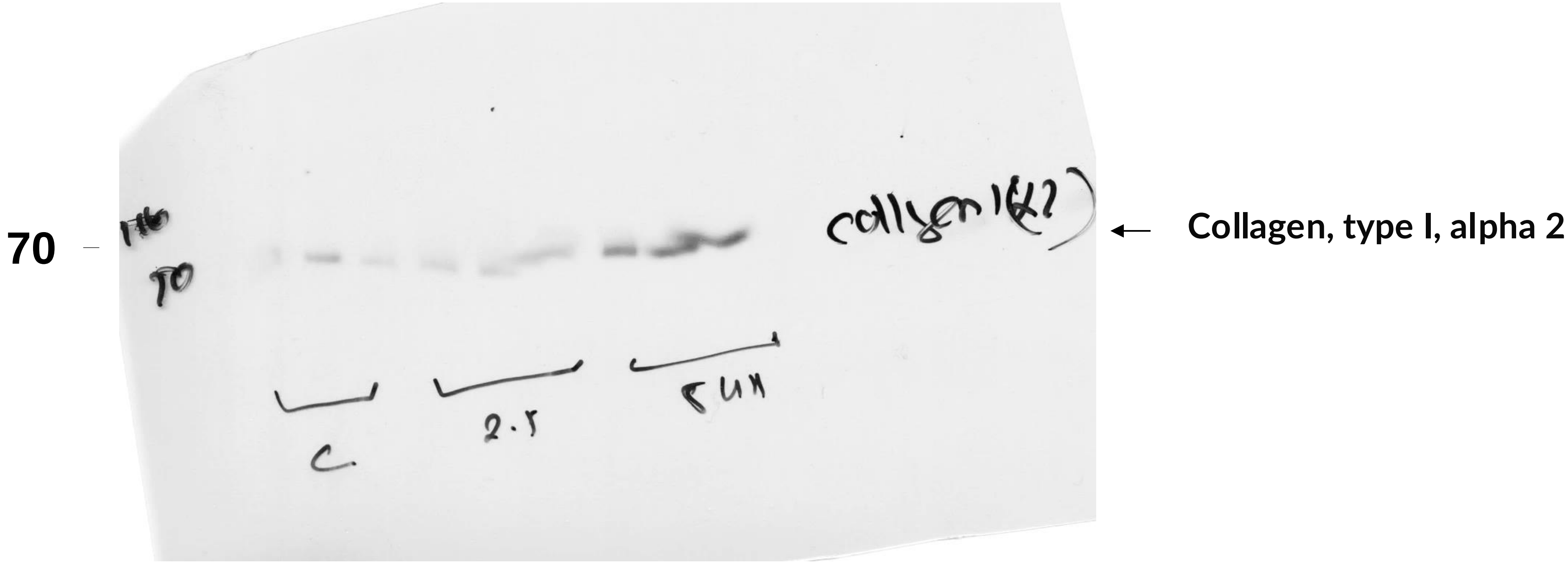

Primary antibodies: mouse monoclonal IgM κ COL1A2 Antibody (G-4) ; Santa Cruz Biotechnology, # sc-166865; 1:1000

Secondary antibodies (Horseradish peroxidase–conjugated secondary antibody (1:10,000))

ECL reagent was used for detecting the protein signal by exposing it to X-ray film in a dark room to visualize the specific protein band recognized by the primary antibody

Full unedited gel for Supplemental Figure 13A. Blot 3/3

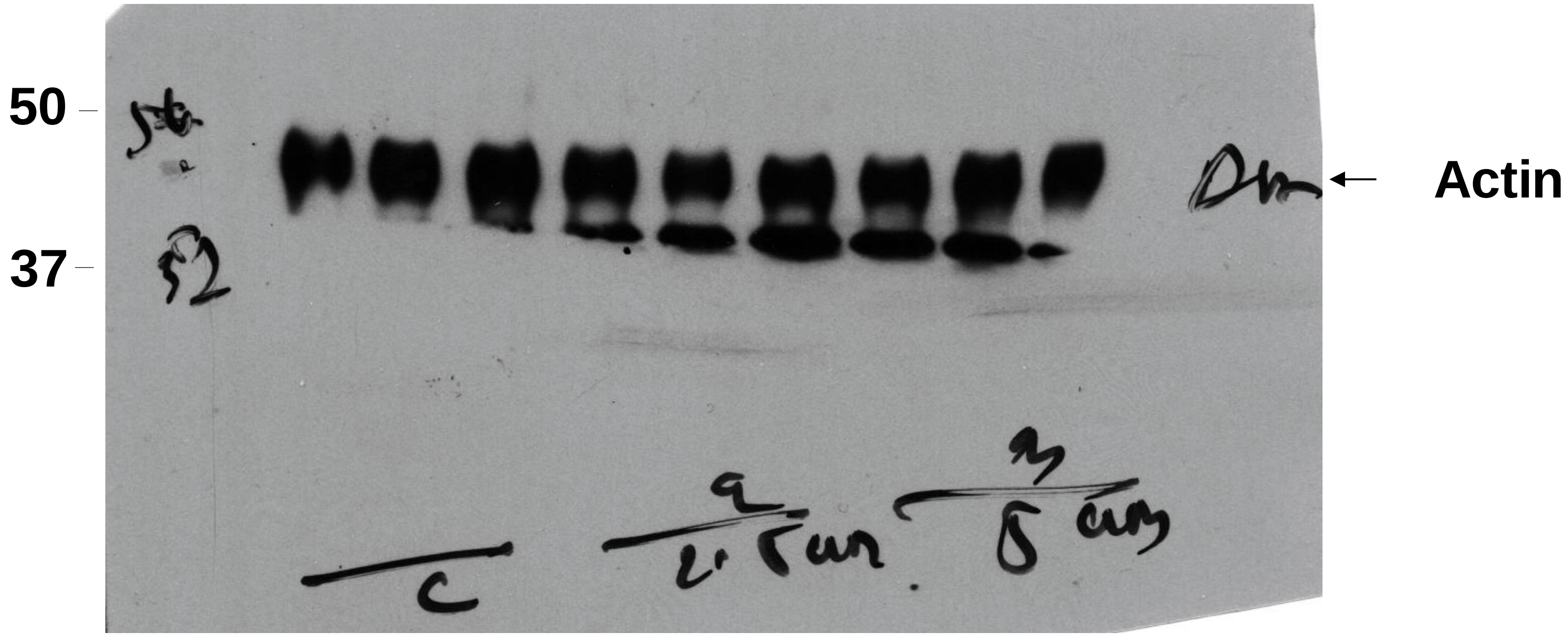

Primary antibodies: Mouse monoclonal IgG1 κ Actin antibody; Santa Cruz Biotechnology, #sc-8432; 1:1000

Secondary antibodies: Horseradish peroxidase–conjugated secondary antibody (1:10,000)

ECL reagent was used for detecting the protein signal by exposing it to X-ray film in a dark room to visualize the specific protein band recognized by the primary antibody
